# Thermal‐Stable and High‐Performance Organic Nonlinear Optical Chromophores for Advanced Electro‐Optic Modulators

**DOI:** 10.1002/advs.202518647

**Published:** 2025-11-16

**Authors:** Changqing Ge, Fuyang Huo, Jiangyi Liu, Xiuyou Han, Zhihua Li, Zhuo Chen, Abdul Rahman, Ruonan Li, Pengwei Li, Shuhui Bo

**Affiliations:** ^1^ Optoelectronics Research Centre Engineering Research Centre of Photonic Design Software (Ministry of Education) School of Science Minzu University of China Beijing 100081 P.R. China; ^2^ Key Laboratory of Bio‐inspired Materials and Interfacial Science Technical Institute of Physics and Chemistry Chinese Academy of Sciences Beijing 100190 P.R. China; ^3^ School of Optoelectronic Engineering and Instrumentation Science Dalian University of Technology Dalian 116024 P.R. China; ^4^ Institute of Microelectronics Chinese Academy of Sciences Ministry of Education Beijing 100029 P.R. China; ^5^ Institute of National Security Mintu University of China Beijing 100081 P.R. China

**Keywords:** electro‐optic coefficient, electro‐optic modulator, high‐speed, low half‐wave voltage, neat chromophores

## Abstract

To address the challenge of developing organic second‐order nonlinear optical materials that combine high electro‐optic (EO) coefficients with excellent thermal stability, four novel chromophores (designated BHG1 to BHG4) are designed and synthesized. These chromophores feature two types of steric hindrance groups—tert‐butyldimethylsilyl (TBDMS)‐bisphenol fluorene and pentafluorobenzyl (PB)‐bisphenol fluorene—into the aniline donor architecture. The incorporation of these bulky substituents not only substantially elevates the glass transition temperatures (T_g_ >130 °C) of all four chromophores but also markedly enhances their long‐term alignment stability. Among them, BHG2 and BHG4, functionalized with a strongly electron‐withdrawing trifluoromethyl‐tricyanofuran (CF_3_‐TCF) acceptor, demonstrate exceptional propensity for self‐assembling into a high‐quality thin film. The neat chromophore BHG2 achieves a remarkable EO coefficient of 304 pm V^−1^, which is attributed to the suppressed dipole–dipole interactions. A high‐speed electro‐optic modulator has been fabricated using the neat BHG2 exhibiting a low half‐wave voltage‐length product (V_π_L) of 2.80 V mm and a 3 dB bandwidth exceeding 40 GHz. These results underscore the significant potential of these materials for advanced optoelectronic devices.

## Graphical abstract

To address the challenge of high electro‐optic (EO) activity and thermal stability, four chromophores (BHG1–BHG4) are designed. These incorporate steric hindrance groups into the aniline donor, significantly raising their glass transition temperatures and enhancing alignment stability. The neat BHG2 film achieves an exceptional EO coefficient of 304 pm V^−1^ and enables a high‐speed modulator with a low V_π_L and a wide bandwidth.

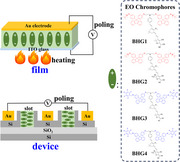

## 1 Introduction

Development and advancement of technologies such as 6G communication, high‐performance computing, microwave photonics, and the Internet of Things, information capacity has grown exponentially over the past decade.^[^ ^1^ ^]^ This rapid growth in technology has placed a significant impact on optical communication systems in terms of bandwidth and energy consumption.^[^ ^2^, ^3^ ^]^ In medium‐ and short‐distance optical communication, achieving ultra‐high‐speed signal transmission has become a hot issue in the IT industry. However, the bandwidth limitation of optoelectronic devices hinders further development and has become a big challenge. At the core of these devices lies the electro‐optic (EO) modulator, responsible for converting electronic signals onto optical carriers.^[^ ^4^ ^]^ The bandwidth and driving voltage of the EO modulator are critical factors that directly impact the information system's bandwidth and energy efficiency.^[^ ^5^, ^6^, ^7^ ^]^ EO materials are essential components of EO modulators. Early research on nonlinear optical materials primarily focused on inorganic crystals and semiconductor materials, such as LiNbO3, InP, and Si, which form the basis of many traditional modulators.^[^ ^8^, ^9^, ^10^, ^11^ ^]^ However, these materials generally exhibit low EO coefficients (e.g., LiNbO_3_ is ≈30 pm V^−1^), leading to higher half‐wave voltages and increased power consumption. In contrast, organic second‐order nonlinear optical (NLO) materials offer numerous advantages, including higher EO efficiency, faster response times, easier processing and integration, and lower costs.^[^ ^12^, ^13^, ^14^, ^15^ ^]^ A standout feature of these organic materials is their tunability through molecular design, enabling optimization of material properties. Research groups have successfully developed high‐performance modulators using organic electro‐optic (OEO) materials, including a 500 GHz EO modulator and even terahertz devices.^[^ ^4^, ^16^, ^17^, ^18^, ^19^, ^20^ ^]^ These achievements highlight the immense potential of OEO materials for future optical communication technologies, and emerging applications, such as LiDAR and optical computing, also require more meticulous consideration of material properties during the development and implementation of new OEO materials.^[^ ^21^ ^]^

OEO materials typically comprise two fundamental components: a polymer matrix and chromophores.^[^ ^22^ ^]^ Chromophores, which are primarily responsible for determining the EO coefficient, generally consist of an electron donor (D), an electron bridge (*π*), and an electron acceptor (A). The first‐order hyperpolarizability (*β*) can be substantially enhanced by an optimized combination of a strong donor, a robust acceptor, and an effective electron bridge. Traditionally, chromophores are either doped into or integrated within a polymer host at concentrations ranging from 20–40wt.%.^[^ ^23^ ^]^ However, recent research has demonstrated remarkable EO performance using neat OEO materials, where chromophores are employed without a polymer matrix, exhibiting very high EO coefficients.^[^ ^24^ ^]^

These neat materials, characterized by high chromophore concentrations, exhibit significant EO activity (r_33_>200 pm V^−1^ in bulk), which translates into superior micro/nano device performance because of 100% active component without a polymer matrix.^[^ ^25^, ^26^ ^]^ Despite these advancements, neat OEO materials often suffer from a low glass‐transition temperature (T_g_<≈85 °C), which limits their thermal stability. For example, the widely studied JRD1 molecule has a T_g_ of ≈82 °C.^[^ ^27^ ^]^ Effective alignment of chromophore dipole moments is essential for achieving EO activity, and this alignment is typically induced through electric field poling at temperatures near T_g_. However, reheating the material to or near its T_g_ often results in the loss of molecular order and EO activity.^[^ ^28^ ^]^ It is well‐documented that EO activity relaxation kinetics are governed by the difference between the measurement temperature (T) and T_g_, expressed as T‐T_g_, and relaxation speeds up when T gets closer to T_g_.^[^ ^29^, ^30^ ^]^

To mitigate this issue, two primary strategies have been investigated: utilizing cross‐linking neat chromophores to form a robust 3D covalent network during the poling process (e.g., HLD1‐2).^[^ ^31^ ^]^ However, these methods often introduce significant complexity into the preparation and poling stages, requiring both pre‐ and post‐crosslinking steps. Additionally, prolonged reaction times can lead to chromophore degradation, ultimately reducing the EO coefficient. Thus, the development of novel methodologies to directly increase the T_g_ of chromophores remains a key objective. A higher Tg neat chromophore would enhance thermal stability, laying the foundation for more efficient and reliable EO materials and devices. Nevertheless, few have succeeded. It demands precise molecular engineering and great expertise to balance high‐quality film formation (flexibility) and a high T_g_ (rigidity).

**Figure 1:**
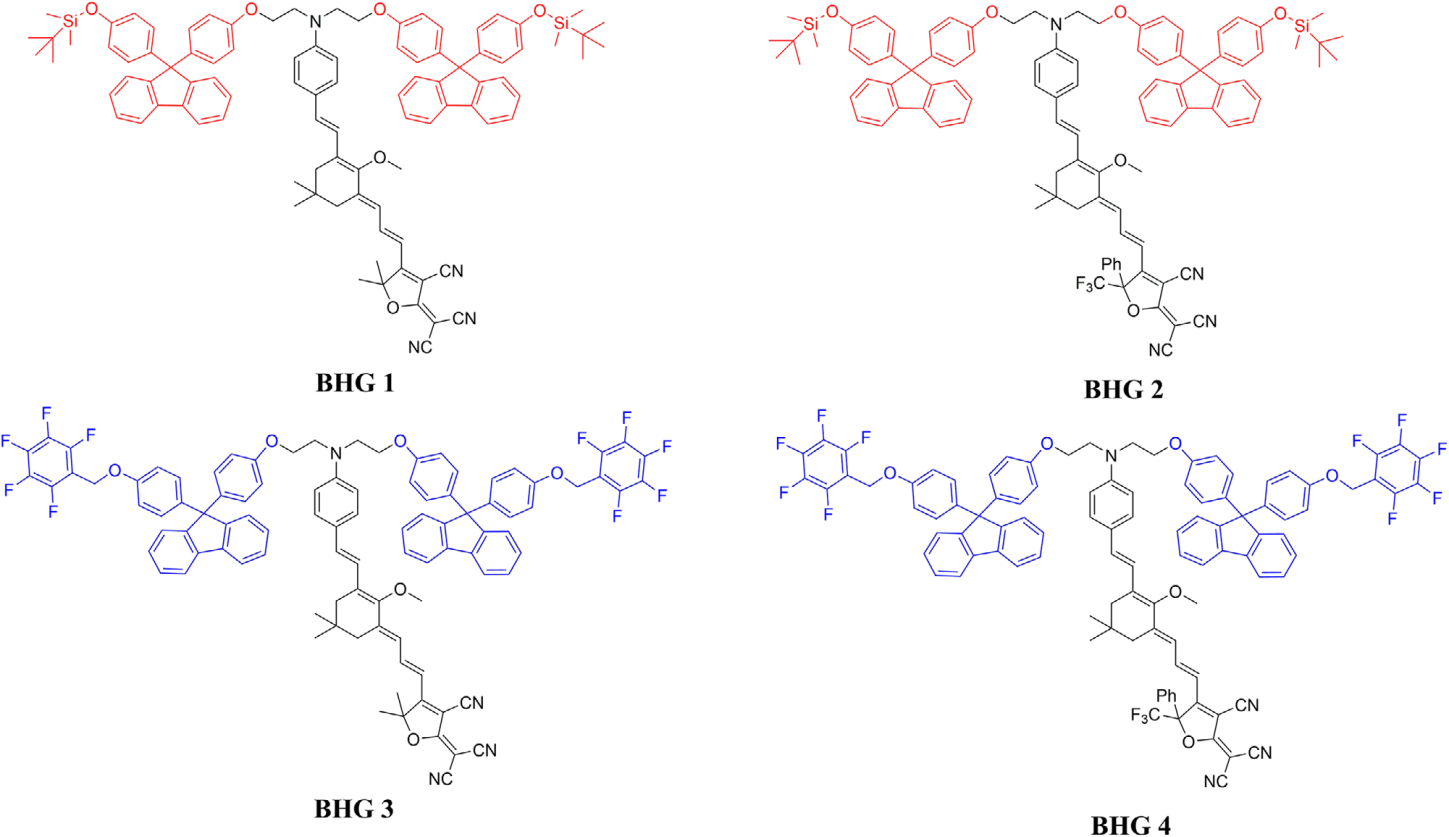
The chemical structure of chromophores BHG1‐4.

In this work, we designed the chromophores BHG1‐BHG4 functionalized with special rigid and bulky modified bisphenol fluorene groups, as shown in **Figure** **1**. Aniline‐based donors, substituted isophorone bridges, tricyanofuran (TCF), or trifluoromethyl tricyanofuran (CF_3_‐TCF) derived acceptors are used to construct the D‐*π*‐A system. The modified bisphenol fluorene rigid group was first introduced into the donor to improve the stability of chromophores (increase T_g_), and additionally, soft tert‐butyldimethyl silyl (TBDMS) or pentafluorobenzene (PB) groups were used to modify bisphenol fluorene to optimize solubility and film‐forming properties. In addition, the modification in the donor also improves the large steric hindrance which is beneficial to weaken the dipole–dipole interaction between the chromophore molecules, and this property of chromophores increases the polarization efficiency and improves EO coefficient. A remarkably high T_g_ is 130 °C was recorded for the neat BHG2 film, the highest EO coefficient is up to 304 pm V^−1^, and the poled BHG2 neat film could still maintain more than 88% or 77% of the origin EO coefficient being placed at 60 or 85 °C for 500 h. The high‐quality EO modulator based on the new neat chromophore BHG2 was also fabricated, and the V*π*L of 2.80 V mm and above 3 dB bandwidth of 40 GHz were obtained in this device. These results show that the OEO materials provided for practical application in high‐performance modulators in optical communications.

## 2 Results and Discussion

**Scheme 1:**
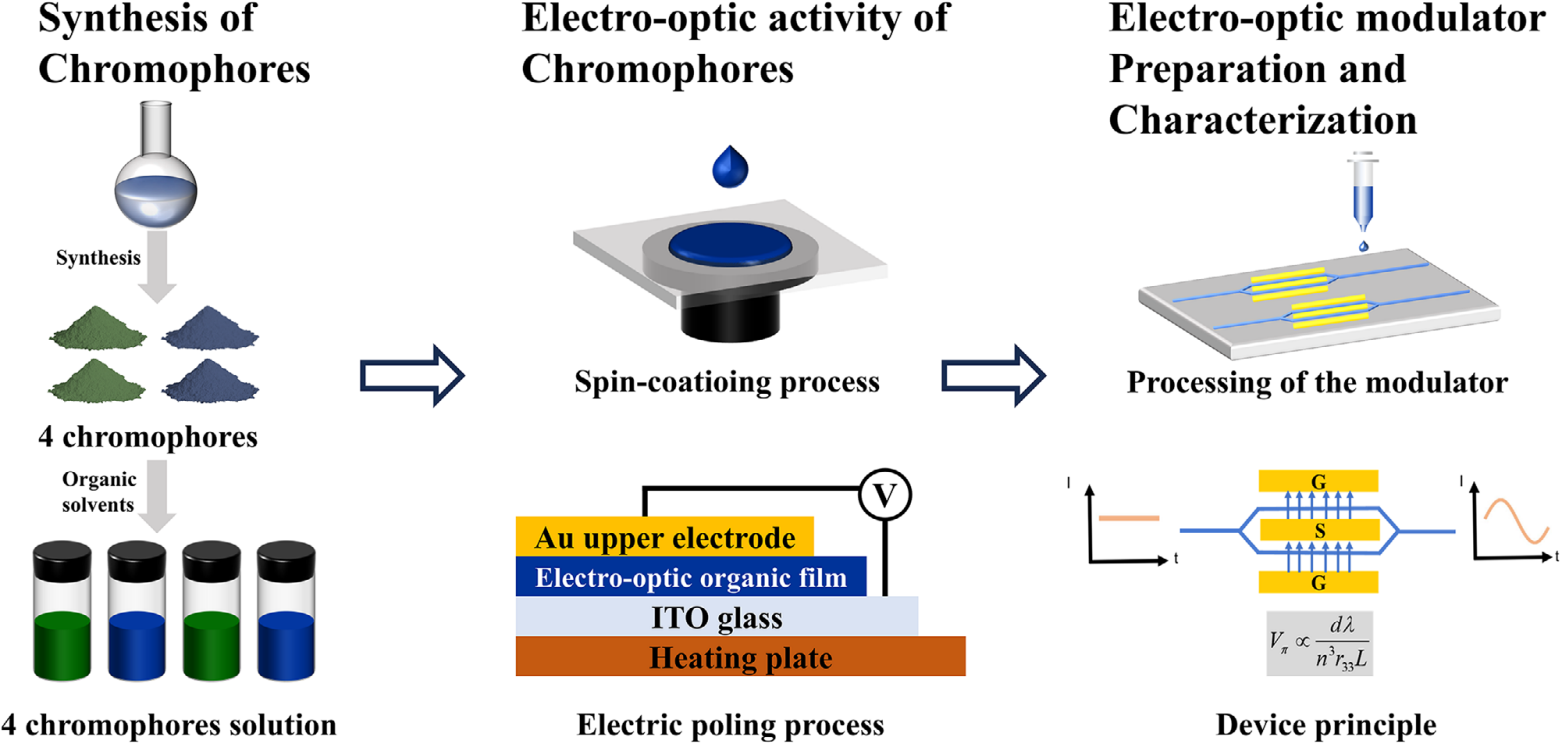
Schematic illustration of overall experimental procedures. (Left) The four chromophores are synthesized, and the powder can dissolve in the organic solvent. (Middle) The films based on the four chromophores are achieved using the spin coating method and poled under the direct current electric field for the OEO materials. (Right) The silicon waveguide EO modulators based on the neat BHG2 chromophore are prepared and characterized.

**Scheme** **1** illustrates the overall experimental layout employed in this research. Initially, four chromophores—BHG1, BHG2, BHG3, and BHG4—were systematically designed and synthesized. BHG1 and BHG2 feature identical aniline donor groups, functionalized with TBDMS‐protected bisphenol fluorene, but differ in their acceptor units. In contrast, BHG3 and BHG4 also possess the same aniline donor groups, modified with PB‐protected bisphenol fluorene, while varying in their acceptor structures. These chromophores were subsequently dissolved in organic solvents, either with or without polymer matrices, and thin films were coated via a spin‐coating technique. The resulting films exhibited EO activity upon application of electric poling. Remarkably, BHG2 demonstrated the capability to form self‐supporting films, making it a promising candidate for EO modulator fabrication. The performance of the fabricated device was then systematically evaluated to assess its electro‐optic properties. The specific synthesis and experimental details are in the Supporting Information.

### 2.1 Synthesis of Chromophores

**Scheme 2:**
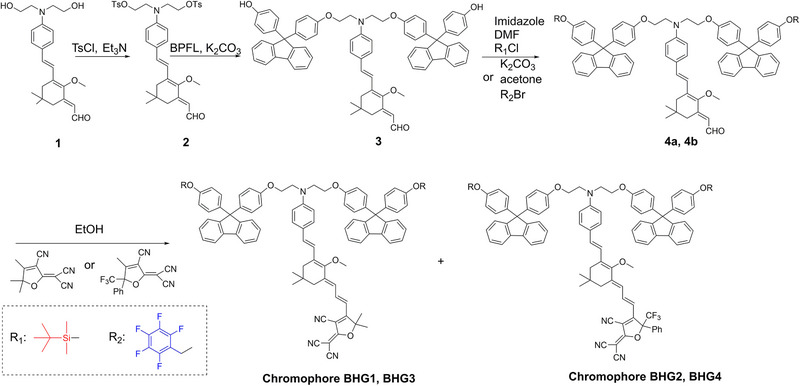
Synthetic routes for chromophores BHG1‐4.

**Scheme** **2** outlines the detailed synthetic pathway and preparation process for these chromophores. The chromophores BHG1‐4 possess functionalized groups that necessitate the presence of two hydroxyl (OH) groups on the donor moiety. Compound 1 was synthesized following a previously reported method.^[^ ^27^ ^]^ The synthesis process of these chromophores involved sequential protection and deprotection of the OH groups. The aldehydes 4a and 4b were condensed with TCF or CF_3_‐TCF acceptor units, resulting in the formation of the green solid chromophores BHG1‐4. The NMR pictures of the chromophores BHG1‐4 have been summarized in Figures S1–S8 (Supporting Information).

### 2.2 Thermal Stability

**Figure 2:**
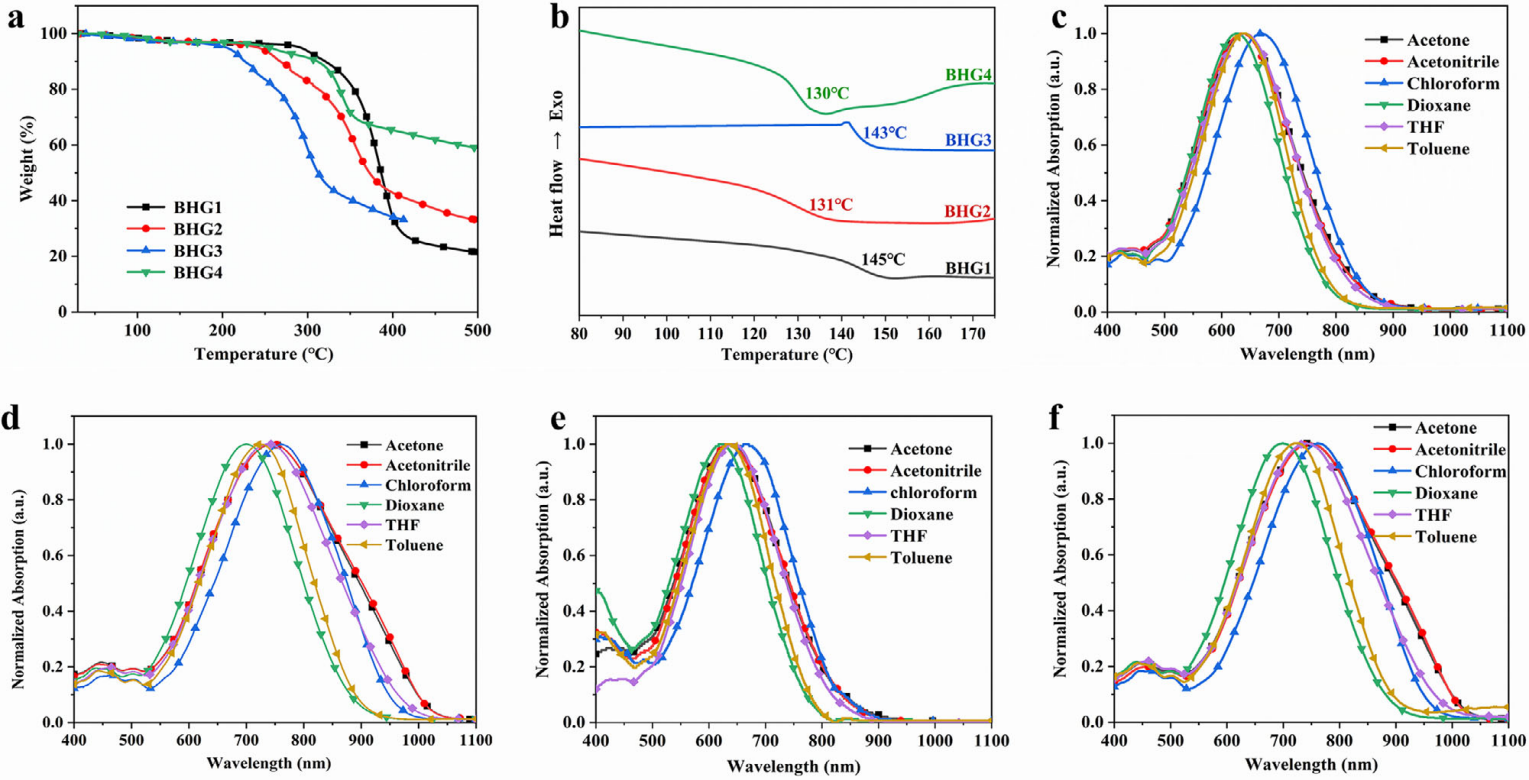
a) TGA curves of chromophore BHG1‐4. b) DSC curves of chromophores BHG1‐4. c) The normalized UV–vis absorption spectra of chromophore BHG1. d) The normalized UV–vis absorption spectra of chromophore BHG2. e) The normalized UV–vis absorption spectra of chromophore BHG3. f) The normalized UV–vis absorption spectra of chromophore BHG4.

In order to obtain a material with macroscopic EO activity, it is necessary to make the dipole molecules in the film undergo a stable directional arrangement. To achieve directional arrangement by means of electric polarization, the material needs to be heated to near the T_g_, so the chromophore molecules must have good thermal stability. The thermal characteristics of the four chromophores were investigated by thermogravimetric analysis (TGA) and differential scanning calorimetry (DSC) as shown in **Figure** **2**a,b and **Table** **1**. The heating rate of 10 °C min^−1^ was set in a nitrogen atmosphere. The T_d_ of BHG1‐4 is 291, 241 °C, 305 °C and 255 °C, respectively. All the chromophores exhibited good thermal stability with T_d_ higher than 240 °C, which is much higher than many chromophores with CF_3_‐TCF.^[^ ^32^ ^]^ The T_g_ of chromophore BHG1‐4 is 145, 131, 143, and 130 °C, respectively. The T_g_ of the four chromophores are higher (≥130 °C), which are more than 50 degrees higher than the previous chromophore (50–85 °C).^[^ ^25^, ^33^, ^34^ ^]^ The high thermal stability of the chromophore results from the introduction of rigid TBDMS‐bisphenol fluorene or PB‐bisphenol fluorene as the steric hindrance groups. Compared with chromophores BHG1 and BHG3, the T_g_ of chromophores BHG2 and BHG4 are lower owing to the CF_3_‐TCF electron acceptor.

**Table 1:** Thermal and optical properties data of the chromophores.

Cmpd	T_d_	T_g_	λ_max_ ^a)^	λ_max_ ^b)^	λ_max_ ^c)^	λ_max_ ^d)^	λ_max_ ^e)^	λ_max_ ^f)^	λ_max_ ^g)^
BHG1	291	145	637	635	670	625	641	638	45
BHG2	241	131	745	747	761	698	738	725	63
BHG3	305	143	635	638	667	621	643	634	46
BHG4	255	130	746	748	761	699	741	723	62

The thermal decomposition temperature T_d_ is the temperature at which the mass of the chromophore sample is reduced to 95 % of the mass before measurement.

^a–f)^ (nm) were the maximum absorption wavelengths in acetone, acetonitrile, chloroform, 1,4‐dioxane, tetrahydrofuran (THF), and toluene, respectively.

^g)^ (nm) was the difference between λ_max_^c)^ and λ_max_^d)^.

### 2.3 Optical Properties

The UV–vis absorption spectra of four chromophores (c = 1 × 10^−5^ mol L^−1^) in six different polar solvents such as acetone, acetonitrile, chloroform, 1,4‐dioxane, tetrahydrofuran, and toluene were tested, as shown in Figure 2 and Table 1. The different polarity of solvents can be determined by their dielectric constant. The dielectric constants at room temperature are 21.0 for acetone, 37.5 for acetonitrile, 4.8 for chloroform, 2.2 for 1,4‐dioxane, 7.6 for tetrahydrofuran, and 2.4 for toluene. The absorption coefficient of four chromophores in six solvents are shown in Table S1 (Supporting Information). The chromophores BHG1‐4 exhibit broad absorption bands, which are attributed to *π*–*π* intramolecular charge transfer (ICT). The solvatochromic behavior of four chromophores in different solvents with different dielectric constants can be studied by UV–vis absorption spectra to explore the polarizability of chromophores.

The maximum absorption peaks (λ_max_) of chromophore BHG1‐4 in chloroform were 670, 761, 667, and 763 nm, respectively. Compared with chromophores BHG1 and BHG3, λ_max_ of the chromophores BHG2 and BHG4 with CF_3_‐TCF acceptor showed a larger redshift, indicating that the introduction of stronger acceptors can significantly enhance the conjugated structure of chromophores and the electron delocalization. The absorption wavelengths of chromophores BHG1 and BHG3 with different steric hindrance groups are almost the same, and it suggests that the rigid steric hindrance group has little effect on the conjugated structure and ICT. At the same time, this also applies to chromophores BHG2 and BHG4.

As shown in Figure 2, the Δλ_max_ (the difference between the maximum absorption wavelengths of chloroform and dioxane) of chromophore BHG1‐4 reached 45, 63, 46, and 62 nm, respectively. The four chromophores show an obvious positive solvatochromic effect. Among them, chromophores BHG2 and BHG4 with CF_3_‐TCF strong acceptor show larger Δλ_max_ than BHG1 and BHG3, indicating that BHG2 and BHG4 have good polarizability and are more sensitive to the external environment.

### 2.4 Theoretical Calculations

In order to further study the influence of the structure of the chromophore on the charge transfer ability and the microscopic nonlinearity, the optimized structure, the first‐order hyperpolarizabilities, dipole moment, and HOMO‐LUMO energy gap of the four chromophores BHG1‐4 (assuming trans‐structure) were calculated by density functional theory (DFT). The calculation software was Gaussian 09, and the parameters were calculated using the CAM‐B3LYP method combined with 6–31 g (d, p) basis group. The data obtained are shown in **Table** **2**.

**Table 2:** DFT calculation data of chromophores BHG1‐4.

Cmpd	E_HOMO_ [eV]	E_LUMO_ [eV]	ΔE [eV]	β_max_ (10^−30^ esu)	µ(D)
BHG1	−5.99	−2.72	3.27	643	24.60
BHG2	−6.06	−2.83	3.23	747	26.32
BHG3	−5.95	−2.69	3.26	592	25.11
BHG4	−6.08	−2.85	3.23	668	26.48

The HOMO‐LUMO energy gap (ΔE) of chromophores BHG1‐4 ranges from 3.23 to 3.27 eV. The energy gap of four chromophores is consistent with the results of the UV–vis absorption spectrum. The energy gap of chromophores BHG2 and BHG4 containing CF_3_‐TCF acceptors is smaller than that of chromophores BHG1 and BHG3 containing TCF acceptors, and the electrons in chromophores BHG2 and BHG4 are more prone to transition. Chromophores BHG1(BHG2) and BHG3(BHG4) show almost the same energy gaps, which suggests that the rigid groups have little effect on the conjugated structure of the chromophores.

In addition, the first‐order hyperpolarizability *β* of the theoretical microscopic static molecules in vacuum was calculated in Table 2. The *β* values of chromophore BHG1‐4 were 643 × 10^−30^, 747 × 10^−30^, 592 × 10^−30^ and 668 × 10^−30^ esu, respectively. According to the two‐level model, the *β* value is related to the chemical structures and the HOMO‐LUMO energy gap. The larger the *β* value is, the stronger the micro nonlinearity of the chromophore is. The order of the *β* value of the four chromophores: BHG2 > BHG4 > BHG1 > BHG3, indicating that the strong electron‐withdrawing ability of the CF_3_‐TCF acceptor is favorable to increase the *β* value of the chromophore, which is consistent with a small energy gap. Strong CF_3_‐TCF electron acceptors also lead to large dipole moments in chromophores BHG2 and BHG4. Meanwhile, the *β* value of chromophore BHG2 is bigger than BHG4, and the *β* value of chromophore BHG1 is greater than BHG3, which suggests that the electron‐donating ability of TBDMS‐bisphenol fluorene may be stronger than PB‐bisphenol fluorene.

The ground state electronic structure of a dipolar molecular could be indicated directly by the frontier molecular orbitals. Figure S9 (Supporting Information) showed the HOMO and LUMO energy level in vacuum of the four chromophores. It could be concluded that all the chromophores showed good planarity, which benefited to the intramolecular electron delocalization. The electron cloud of HOMO is mainly localized on the *π* electron bridge and the donor, while the electron cloud of LUMO is mainly localized on the TCF or CF_3_‐TCF acceptor group and the *π* electron bridge. The electron cloud was more concentrated in the acceptor of the chromophores with the strong acceptor CF_3_‐TCF than the chromophores with the acceptor TCF. This was because of the stronger electron withdrawing ability of the CF_3_‐TCF acceptor. There is nearly no electron cloud dense distribution in the rigid steric group region, and there is no significant contribution to the intramolecular charge transfer, which is confirmed in the results of the UV–vis absorption spectrum.

### 2.5 Electro‐Optic Properties (r_33_)

To study the macroscopic EO activity of the four chromophores, it is necessary to investigate the self‐forming films properties of the chromophores. The picture of the four chromophores after self‐forming films with a 200‐fold optical microscope are shown in Figure S10 (Supporting Information). The neat chromophores BHG2 and BHG4 have good film‐forming properties (the roughness of film listed in Table S2 and Figure S11, Supporting Information), while the neat chromophores BHG1 and BHG3 have poor film‐forming properties. If the film is thick, cracks will occur. This is on the basis that acceptor CF_3_‐TCF is more favorable for film forming than TCF.

To optimize the poling conditions and measurement of EO activity, thin‐film devices of materials sandwiched between ITO and gold were prepared for poling and r_33_ measurement by the Teng‐Man technique.^[^ ^35^ ^]^ Chromophores BHG1‐4 were doped into Polymethyl methacrylate (PMMA) to obtain the thin film, and the doping concentration was 25 wt.% (BHG1/PMMA, BHG2/PMMA, BHG3/PMMA and BHG4/PMMA). The thickness of the doped PMMA films obtained by spin‐coating is ≈2–3 µm. The neat chromophores BHG2 and BHG4 with the mass fraction of 8–10 wt.% dissolved in 1,1,2‐trichloroethane were used as the solution for spin‐coating to obtain a film thickness between 1.0 and 1.3 µm. After the film is prepared, 50 nm thick gold electrodes are sputtered, and then the poling process is performed.

When poling, the film was heated at a suitable temperature (5–10 °C higher than the T_g_ of the chromophore) for contact poling under a nitrogen atmosphere, and then a DC electric field (E_p_ is ≈20–120 V µm^−1^ for BHG/PMMA, and E_p_ is ≈20–60 V µm^−1^ for the neat BHG2 and BHG4) was applied to induce acentric order in the chromophores. After the poling process is completed, the electro‐optic film cools to room temperature. The electro‐optic coefficient (r_33_) of the poling OEO film was measured by the simple reflection method using carefully selected thin ITO glass as the lower electrode with low reflectivity and good transparency to minimize the influence from multiple reflections. The average poling efficiency r_33_/Ep of multiple poling experiments of different samples was calculated from the slope of the r_33_ vs average poling field plot in **Table** **3** and Figure S12 (Supporting Information).

**Table 3:** The maximum r_33_ value of the chromophore and the corresponding poling data.

Cmpd	ρ_N_ ^b)^	poling temp [°C]	r_33_/E_p_ [nm^2^ v^−2^]^c)^	r_33_/[E_p_ρ_N_]^d)^	Max. r_33_ [pm V^−1^]^e)^
BHG1	4.13	N/D	N/D	N/D	N/D
BHG2	3.82	138	5.53 ± 0.6	1.45 ± 0.2	304
BHG3	3.79	N/D	N/D	N/D	N/D
BHG4	3.53	136	3.40 ± 0.7	0.96 ± 0.2	221
^a)^BHG1/PMMA	1.03	107	1.30 ± 0.1	1.26 ± 0.1	117
^a)^BHG2/PMMA	0.95	102	2.18 ± 0.4	2.29 ± 0.4	193
^a)^BHG3/PMMA	0.95	105	0.78 ± 0.1	0.82 ± 0.1	84
^a)^BHG4/PMMA	0.88	99	1.72 ± 0.2	1.95 ± 0.2	153

^a)^ with 25 wt.% content chromophores in PMMA polymer.

^b)^ Chromophore number density; in units of ×10^20^ molecules per cc.

^c)^ Average from multiple poling experiments.

^d)^ Poling efficiency per number density (nm^2 ^V^−2^(10^20^ molecules cm^−3^)^−1^).

^e)^ Experimental r_33_ values from simple reflection at 1310 nm.

The EO coefficient value of the chromophores depends on the number density of the chromophore (N), the orientation parameter (<cos3θ>), and the first‐order hyperpolarizability (*β*) of the chromophore molecules.^[^ ^36^, ^37^ ^]^ The relationship is as follows:

(1) $$r_{33}=\left|\frac{2Nf\left(\omega \right)\beta \langle \cos^{3}\theta \rangle }{n^{4}}\right|$$

ƒ(ω): Lorentz–Onsager local area field factor, depending on the medium characteristics of the surrounding environment of the chromophore; n: refractive index of the material; *θ* refers to the angle between the dipole moment of the chromophore and the direction of the electric field. The orientation sequence parameter reflects the efficiency of the directional arrangement of the chromophore during the poling process. However, chromophore molecules have significant dipole–dipole interactions, which can hinder the molecular rotation under the electric field. So the poling efficiency of chromophores can be effectively improved by introducing steric hindrance groups into chromophores.^[^ ^38^ ^]^

Through the experimental data in Table 3, the maximum macroscopic EO coefficient of the chromophore is consistent with the trend of the microscopic first‐order hyperpolarizability *β*, and the order of the maximum r_33_ value is BHG2/PMMA > BHG4/PMMA > BHG1/PMMA > BHG3/PMMA, which is attributed to BHG2 and BHG4 with the stronger acceptor than BHG1 and BHG3. Compared with electro‐optic film BHG3/PMMA (84 pm V^−1^), the BHG1/PMMA showed a higher electro‐optic coefficient (117 pm V^−1^), and the same situation also occurred in BHG2/PMMA (193 pm V^−1^) and BHG4/PMMA (153 pm V^−1^). The neat chromophore BHG2 has a higher EO coefficient of 304 pm/V than the neat chromophore BHG4. The above results indicate that the electron‐donating ability or steric hindrance effect of TBDMS‐bisphenol fluorene is stronger than that of PB‐bisphenol fluorene. The TBDMS‐bisphenol fluorene group may effectively weaken dipole–dipole interactions between chromophores and improve the poling efficiency.

After comparing the data, it is found that the maximum EO coefficients of the neat chromophore BHG2 and BHG4 are generally bigger than those of chromophore BHG2/PMMA and BHG4/PMMA, respectively. On the one hand, the number density N of the neat chromophore BHG2 and BHG4 ordered orientation chromophore is much higher than that of chromophore BHG2/PMMA and BHG3/PMMA. On the other hand, TBDMS‐bisphenol fluorene or PB‐bisphenol fluorene as the steric hindrance group can weaken dipole–dipole interactions to enhance the poling efficiency per number density of the neat chromophores. The average r_33_/E_p_ values of BHG1/PMMA, BHG2/PMMA, BHG3/PMMA, BHG4/PMMA, BHG2 and BHG4 were 1.30 ± 0.1, 2.18 ± 0.4, 0.78 ± 0.1, 1.72 ± 0.2, 5.53 ± 0.6 and 3.40 ± 0.7 nm^2^ V^−2^, respectively. The poling efficiency of the neat chromophores far exceeds that of BHG/PMMA, which indicates that the neat chromophores help to improve the EO activity. Poling efficiency per number density of the neat chromophores is relatively low, and this may be solved through better molecular optimization design and withstanding higher poling voltage strength.

Table 3 summarizes the properties of the reported state‐of‐the‐art neat EO chromophores. Comparable to state‐of‐the‐art values of the previously reported organic EO chromophores, the BHG2 and BHG4 possess the highest T_g_, several tens of degrees higher than other chromophores, indicating excellent high‐temperature thermal stability of r_33_. Meanwhile, the neat BHG2 chromophore exhibits a high EO coefficient. To investigate the stability of the neat chromophores, and long‐term thermal stability of BHG2 was tested to achieve practical and commercial applications in Figure S13 (Supporting Information). JRD1, a famous chromophore with a high EO coefficient and T_g_ of ≈82 °C,^[^ ^27^ ^]^ was also tested for the long‐term thermal stability for the reference. The neat BHG2 chromophore film could still maintain more than 88% of the original EO coefficient being placed at 60 °C for 500 h, while the neat JRD1 chromophore only remains 43%. When the curing temperature rises to 85 °C for 500 h, the neat BHG2 chromophore film could still maintain more than 77%, but the neat JRD1 chromophore only remains 15% at the first 24 h. The above results indicate that the high glass transition temperature of BHG2 is beneficial for the long‐term alignment stability of the chromophores. Hence, the presented BHG series chromophores stand out in both EO efficiency and high‐temperature stability (**Table** **4**).

**Table 4:** Properties of the state‐of‐the‐art neat chromophores with CF_3_‐TCF acceptor.

Cmpd	T_d_ [℃]	poling temp [°C]	r_33_ [pm V^−1^]
JRD1^[27]^	226	82	343 ± 55
YLD124^[^ ^32^ ^]^	208	81	242
QLD1^[^ ^39^ ^]^	273	93	252
QLD2^[^ ^40^ ^]^	317	75	233
QLD3^[^ ^40^ ^]^	270	74	242
HLD1^[^ ^31^ ^]^	231	75	218±9
HLD2^[^ ^28^ ^]^	317	72	200 ± 10
FZL1^[^ ^37^ ^]^	274	68	180
FZL2^[^ ^37^ ^]^	298	70	223
FZL3^[^ ^37^ ^]^	287	64	159
FZL4^[^ ^37^ ^]^	307	70	127
BHG2^a)^	241	131	304
BHG4^a)^	255	130	221

^a)^ This work.

### 2.6 Electro‐Optic Modulator Preparation and Characterization

The material's behavior, including its state and polarization field, are vastly different during the above processes compared to in the final device. So the EO modulators based on the neat BHG2 were first prepared for broadband optical communication and microwave photonic technologies. Silicon‐based photonics is a relatively mature process platform, and organic EO materials precisely have good integration and compatibility. By combining the high confinement optical capability of silicon‐based optical waveguides with the high EO coefficient of organic EO materials, it is expected to achieve low drive, large bandwidth, and small‐sized electro‐optic modulators.

**Figure 3:**
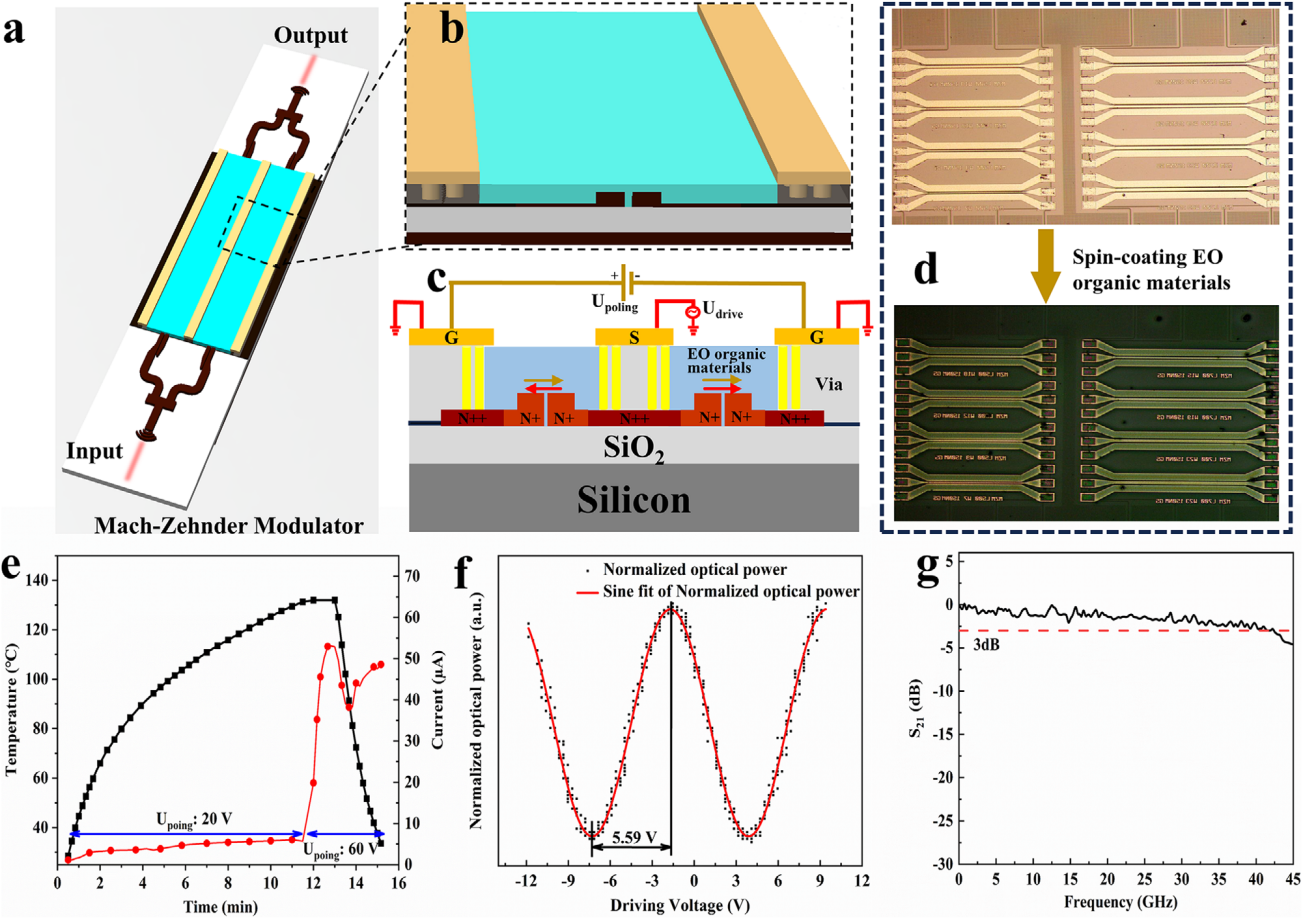
a) A schematic of MZI modulator with the optical input signal and output signal through silicon grating coupler. b) The partially enlarged detailed image of the a). c) Cross‐section schematic diagram of optical waveguide electro‐optic modulator. We use G‐S‐G electrode system, with two ground (G) electrodes for polarization, the signal electrode (S) in the middle, and the ground electrode (G) on both sides, which enables push‐pull driving. d) Spin coating OEO materials on silicon‐based waveguide devices for heterogeneous EO modulators. e) Optimal electric poling conditions curve, including poling temperature, time, voltage, and temperature (the black line is temperature, and the red line is current). f) The output optical signal (P) and driving voltage (V) curve. g) The measured EO S_21_ of the modulator with the modulation length of 500 µm.

The refractive index n and extinction coefficient k of the neat BHG2 film before and after poling are very important parameters for the design of optical waveguide devices, which are tested by an ellipsometer in the wavelength range of 400–1700 nm and shown in Figure S14 and Table S3 (Supporting Information). The EO modulator has been prepared as previously published article, and the structure of the devices is as shown in **Figure** **3**a.^[^ ^41^ ^]^ In the Mach‐Zehnder interferometer (MZI) modulator, a laser with a wavelength of 1550 nm enters the silicon strip waveguide from the grating coupler, and splits into two via the multimode coupler, and then goes into the slot waveguide through the self‐designed strip‐to‐slot mode convertor. Optical carrier merges through the multimode coupler again, and is output through the grating coupler. In Figure 3b,c, the width of the slot is only 150 nm. To ensure conductivity for poling and driving process, the silicon slab waveguide is connected to the slot waveguide using partition doping. The silicon slab near the slot is n‐doped with a concentration of 3 × 10^16^ cm^−3^ (N+) with less optical loss, while the concentration farther away is 1 × 10^20^ cm^−3^ (N++) to achieve better conductivity. By spin coating BHG2 onto silicon‐based waveguide devices, a heterogeneous integrated electro‐optic modulator is completed in Figure 3d.

The poling process for optimizing conditions (voltage, current, temperature, and time) needs to be executed to achieve effective modulation efficiency. At T_g_, the BHG2 chromophores with a big dipole moment tend to align with the electric field direction, and the alignment will be maintained when the material is cooled to room temperature to achieve a macroscopic EO effect. The poling voltage was added on the two ground electrodes (The poling direction is marked by the golden yellow arrow), and the voltage was added on the signal electrode and ground electrodes for push‐pull drive (The drive direction is marked by the red arrow) in Figure 3c. Figure 3e shows the optimized poling conditions on the chip.

The half‐wave voltage directly reflects the modulation efficiency of the electro‐optic modulator. An EO modulator with a smaller half‐wave voltage means it can achieve larger phase modulation at lower voltages, resulting in higher modulation efficiency and lower energy consumption. The half‐wave voltage V_π_ of the modulator is measured by the driving voltage of a triangle wave signal, and the result is shown in Figure 3f. The measured half‐wave voltage V_π_ at 100 kHz is 5.59 V, and the half‐wave voltage length product V_π_L is 2.80 V mm, with the modulation length of 500 µm. The value of V_π_L is only one tenth of the reported modulator in the recent published literature about TFLN (Thin‐film lithium niobate) modulator.^[^ ^42^, ^43^ ^]^

The frequency response bandwidth is a key indicator of a modulator, which determines the frequency range the modulator can work. The bigger bandwidth of the modulator means an increment in the amount of data that can be transmitted, thereby improving communication efficiency. To accurately estimate the 3 dB bandwidth of the modulator, the EO S_21_ curve was measured using an electrical vector network analyzer (VNA). The bandwidth of the probe we use is DC to 40 GHz, and the S_21_ curve decreases by 3 dB as shown in Figure 3g. The cables, RF probes, and photodetectors have all been calibrated, and EO modulator with a 3 dB bandwidth is over 40 GHz. Testing setup for the EO frequency response of the modulator including electrical connections (probe) and optical paths using single‐mode fibers in Figure S15 (Supporting Information). If a higher frequency probe is used, the 3 dB bandwidth of the modulator will be larger, as our previous work suggests that the device's minimal transmission loss and strong electro‐optic activity are conducive to achieving such higher frequency performance.^[^ ^41^ ^]^

## 3 Conclusion

In pursuit of achieving both a high EO coefficient and excellent thermal stability in organic NLO materials, we have successfully designed and synthesized four novel chromophores, BHG1–BHG4. These chromophores incorporate two rigid steric hindrance groups: tert‐butyldimethylsilyl (TBDMS)‐bisphenol fluorene and pentafluorobenzyl (PB)‐bisphenol fluorene, marking the first reported use of this combination. By strategically selecting these steric hindrance groups, we achieved a significant increase in the glass transition temperatures of the chromophores, improving thermal stability by several tens of degrees. Notably, BHG2 demonstrated exceptional film‐forming properties and achieved the highest EO coefficient of 304 pm V^−1^ without requiring complex preparation or polarization processes. This effective molecular design approach successfully addresses the dual challenges of achieving both high EO activity and robust thermal stability, making BHG2 highly suitable for device fabrication. Furthermore, we validated the practical applicability of BHG2 by integrating it into a high‐speed EO modulator, combining silicon waveguides with organic materials. The resulting modulator exhibited outstanding EO performance, including a V_π_L of 2.80 V mm and a 3 dB bandwidth exceeding 40 GHz. These findings highlight that the careful design of steric hindrance groups represents a powerful molecular engineering strategy for improving both the EO coefficient and thermal stability of organic EO materials. This approach paves the way for developing highly efficient, large‐area, and low‐loss EO modulators and devices suitable for real‐world high‐speed optical communication systems.

## 4 Experimental Section

The experimental procedures can be found in the Supporting Information.

## Conflict of Interest

The authors declare no conflict of interest.

## Supporting information



Supporting Information

## Data Availability

The data that support the findings of this study are available from the corresponding author upon reasonable request.
