# Supplementary material for: Thermal‐Stable and High‐Performance Organic Nonlinear Optical Chromophores for Advanced Electro‐Optic Modulators
Source: Adv Sci (Weinh). 2025 Nov 16;13(7):e18647. doi: 10.1002/advs.202518647 (PMC12866772; doi:10.1002/advs.202518647)
Supplement: Supplementary file 1 — Supporting Information [file ADVS-13-e18647-s001.docx]

Supporting Information

Thermal-Stable and High-Performance Organic Nonlinear Optical Chromophores for Advanced Electro-Optic Modulators

Changqing Ge, Fuyang Huo, Jiangyi Liu, Xiuyou Han, Zhihua Li, Zhuo Chen*, Abdul Rahman, Ruonan Li, Pengwei Li, Shuhui Bo*

C. Q. Ge, J. Y. Liu, A. Rahman, R. N. Li, P. W. Li, S. H. Bo

Optoelectronics Research Centre, Engineering Research Centre of Photonic Design Software (Ministry of Education), School of Science, Minzu University of China, Beijing, 100081, PR China.

E-mail: boshuhui@muc.edu.cn

F. Y. Huo, Z. Chen

Key Laboratory of Bio-inspired Materials and Interfacial Science, Technical Institute of Physics and Chemistry, Chinese Academy of Sciences, Beijing, 100190, PR China.

E-mail: chenzhuo@mail.ipc.ac.cn

X. Y. Han

School of Optoelectronic Engineering and Instrumentation Science, Dalian University of Technology, Dalian 116024, PR China.

Z. H. Li
Institute of Microelectronics, Chinese Academy of Sciences, Ministry of Education, Beijing, 100029, PR China.

S. H. Bo

Institute of National Security, Mintu University of China, Beijing, 100081, PR China.

E-mail: boshuhui@muc.edu.cn

**1. Materials and instruments**

The chemical reagents used were purchased from Aladdin or Sigma-Aldrich and used directly without further purification unless otherwise stated. The solvents used like tetrahydrofuran (THF) and N,N-dimethylformamide (DMF) were commercial ultra-dry reagents. 2-(3-cyano-4,5,5-trimethylfuran-2(5H)-ylidene)malononitrile (TCF) and 2-(3-cyano-4-methyl-5-phenyl-5-(trifluoromethyl)furan-2(5H)-ylidene)malononitrile (CF_3_-TCF) acceptors were prepared according to the literature.[^1^](#_ENREF_22)

Instruments and equipment: NMR (Advance Bruker 400 M, 600M), TGA (Setline); DSC (TA-DSC 25), UV-visible absorption spectrum (UV-3600 Plus), KW-4B spin-coating machine, Film thickness and film roughness (BRUKER DedtakXT step meter), Refractive index: (Horoba UVISEL PLUS ellipsometer). Arbitrary waveform generator (Agilent 33250A), Oscilloscope (Agilent DSO6032A, 300 MHz), Cascade probes (DC to 40 GHz), VNA (AV3672D, 10 MHz to 50 GHz).

**2. Experimental**

**2.1. Synthesis of compound 2**

Under nitrogen protection, compound 1 (3.85 g, 10 mmol) and p-toluenesulfonyl chloride (TsCl, 5.30 g, 30 mmol) were dissolved in 30 mL of redistilled THF solvent, cooled to 0℃, and triethylamine was added dropwise. The temperature was maintained for 1 h, the ice bath was removed, and the reaction was allowed to proceed overnight at room temperature. The mixture was poured into 50 mL of deionized water, extracted with ethyl acetate three times, and the organic part were combined. Then, the unreacted p-toluenesulfonyl chloride was washed with 10% NaOH solution. The product was dried with anhydrous MgSO_4_ and evaporated to obtain a red solid without separation.

**2.2. Synthesis of compound 3**

A mixture of compound 2 (1.386 g, 2 mmol), Fluorene-9-bisphenol (BPFL, 3.50 g, 10 mmol) and anhydrous potassium carbonate (1.40 g, 26 mmol) were dissolved in 50 mL of DMF solution. Under nitrogen protection, the reaction was heated at 100℃ for 24 h. The mixture was poured into 200 mL of deionized water and extracted with ethyl acetate three times. The organic phases were combined and then washed with saturated NaCl solution. After drying with MgSO_4_, the organic solvent was removed by rotary evaporation, and the product was passed through a silica gel column. A red solid was obtained with a yield of 52%. MS: Calculated 1049.47, found 1049.60.

^1^H NMR (400 MHz, CDCl_3_) δ 10.05 (d, J = 8.3 Hz, 1H), 7.96 (s, 1H), 7.71 (d, J = 7.4 Hz, 4H), 7.36–7.28 (m, 10H), 7.25–7.20 (m, 5H), 7.04 (dd, J = 20.2, 7.9 Hz, 8H), 6.67 (d, J = 8.4 Hz, 11H), 6.31 (d, J = 8.2 Hz, 1H), 4.07 (s, 4H), 3.79 (s, 4H), 3.61 (s, 3H), 2.71 (s, 2H), 2.37 (s, 2H), 1.04 (s, 6H). ^13^C NMR (101 MHz, CDCl_3_) δ 191.44, 162.82, 157.23, 154.88, 151.80, 151.71, 149.65, 147.46, 139.90, 138.66, 137.52, 132.86, 132.55, 132.27, 130.94, 129.25, 129.20, 128.60, 127.64, 127.28, 125.97, 125.72, 121.66, 120.08, 119.95, 115.11, 114.03, 112.00, 65.17, 64.13, 61.08, 51.09, 39.10, 38.80, 36.61, 31.59, 30.71, 28.24.

**2.3. Synthesis of compound 4a, 4b**

Under nitrogen, tert-butyldimethylchlorosilane (3.68 g, 24 mmol) was slowly added to the solution of compound 3 (10.50 g, 10 mmol) and imidazole (1.66 g, 24 mmol) in 20 mL DMF. After stirring at room temperature for 3 h, pour it into 100 mL of water. The organic phase was extracted with n-hexane, washed with salt water, and dried with MgSO_4_. After the solvent was removed by rotary evaporation, the crude product was purified by silica gel column chromatography and eluted with ethyl acetate/hexane (1:15 to 1:10) to obtain the red oily compound 4a, with a yield of 93.1%. HRMS (ESI) (M+H)^+^: calcd for (C_85_H_91_NO_6_Si_2_+H)^+^: 1278.6458; found: 1278.6412.

^1^H NMR (400 MHz, CDCl_3_) δ 10.08 (d, *J* = 8.3 Hz, 1H), 7.71 (d, *J* = 7.5 Hz, 4H), 7.33 (dt, *J* = 14.8, 5.4 Hz, 10H), 7.21 (t, *J* = 7.3 Hz, 5H), 7.05 (dd, *J* = 13.2, 8.7 Hz, 8H), 6.67 (dd, *J* = 8.5, 4.9 Hz, 11H), 6.33 (d, *J* = 8.3 Hz, 1H), 4.05 (t, *J* = 5.6 Hz, 4H), 3.78 (t, *J* = 5.5 Hz, 4H), 3.64 (s, 3H), 2.72 (s, 2H), 2.37 (s, 2H), 1.05 (s, 6H), 0.95 (s, 18H), 0.15 (s, 12H).

The mixture of pentafluorobenzyl bromide (6.79 g, 26 mmol), compound 3 (13.65 g, 13 mmol), potassium carbonate (3.63 g, 26 mmol) and KI (0.33 g, 2 mmol) was dissolved in anhydrous DMF. The reaction mixture was heated at 85 °C for 20 hours. After cooling to room temperature, pour the reaction mixture into water. The mixture was extracted with CH_2_Cl_2_ and dried with MgSO_4_. After the solvent was removed under reduced pressure, the residue was purified by column chromatography to obtain an orange solid product 4b with a yield of 80%. HRMS (ESI) (M): calcd for (C_87_H_65_F_10_NO_6_): 1409.4725; found: 1409.4652.

^1^H NMR (400 MHz, CDCl_3_) δ 10.09 (d, J = 8.2 Hz, 1H), 7.73 (d, J = 7.4 Hz, 4H), 7.33 (t, J = 7.8 Hz, 10H), 7.24 (d, J = 4.3 Hz, 5H), 7.09 (dd, J = 18.1, 7.9 Hz, 8H), 6.79 (d, J = 7.9 Hz, 4H), 6.75 – 6.64 (m, 7H), 6.31 (d, J = 8.2 Hz, 1H), 5.02 (s, 4H), 4.09 (t, J = 5.2 Hz, 4H), 3.82 (s, 4H), 3.63 (s, 3H), 2.73 (s, 2H), 2.38 (s, 2H), 1.04 (d, J = 12.7 Hz, 6H).

**2.4. Synthesis of chromophore BHG1 and BHG3**

Compound 4a or 4b (1.2 mmol) and TCF receptor (0.28 g, 1.4 mmol) were dissolved in 20 mL of absolute dry ethanol, refluxed at 80 ° C for 0.5 h, and the solvent was removed by rotary evaporation. The crude product was purified by silica gel obtain chromophore BHG1 and BHG3 with the yield of 34.1 % and 41.2%. HRMS (ESI) (M+H)^+^ for BHG1: calcd for (C_96_H_98_N_4_O_6_Si_2_+H)^+^: 1459.7098; found: 1459.7062. HRMS (ESI) (M+H)^+^ for BHG3: calcd for (C_96_H_72_F_10_N_4_O_6_+H)^+^: 1591.5365; found: 1591.5342.

^1^H NMR for BHG1 (400 MHz, CDCl_3_) δ 7.85 (m, 1H), 7.58 (d, *J* = 7.5 Hz, 4H), 7.18 (dt, *J* = 11.7, 8.0 Hz, 10H), 7.09 (m, 5H), 6.91 (dd, *J* = 17.5, 8.6 Hz, 8H), 6.64 – 6.49 (m, 12H), 6.22 (d, *J* = 14.9 Hz, 1H), 3.95 (t, *J* = 5.6 Hz, 4H), 3.68 (t, *J* = 5.3 Hz, 4H), 3.55 (s, 3H), 2.33 (d, *J* = 18.4 Hz, 4H), 1.54 (s, 6H), 0.91 (s, 6H), 0.80 (s, 18H), 0.15 (s, 12H). ^13^C NMR for BHG1 (101 MHz, CDCl_3_) δ 176.10, 173.27, 157.19, 154.36, 151.81, 151.63, 148.02, 143.97, 139.97, 138.82, 138.39, 134.56, 133.61, 129.25, 129.14, 128.97, 127.64, 127.32, 126.06, 123.01, 120.44, 120.12, 119.47, 116.75, 114.06, 112.68, 112.47, 111.81, 111.61, 96.91, 94.83, 65.07, 64.23, 61.56, 51.33, 40.14, 39.14, 30.97, 28.35, 26.44, 25.67, 18.14, -4.38.

^1^H NMR for BHG3 (600 MHz, CDCl_3_) δ 7.74 (d, J = 7.6 Hz, 4H), 7.38 (d, J = 8.6 Hz, 2H), 7.33 (t, J = 6.6 Hz, 8H), 7.27 – 7.21 (m, 6H), 7.14 – 7.05 (m, 8H), 6.83 – 6.65 (m, 12H), 6.37 (d, J = 14.8 Hz, 1H), 5.03 (s, 4H), 4.10 (s, 4H), 3.84 (s, 4H), 3.69 (s, 3H), 2.45 (d, J = 25.3 Hz, 4H), 1.69 (s, 6H), 1.06 (s, 6H). ^13^C NMR for BHG3 (151 MHz, CDCl_3_) δ 176.11, 173.24, 157.32, 156.69, 151.52, 148.03, 147.90, 143.96, 139.94, 139.33, 138.41,134.69, 133.74, 129.31, 129.22, 128.98, 127.74, 127.45, 125.92, 125.68, 122.90, 120.21, 116.68, 114.30, 114.08, 111.96, 96.89, 65.17, 64.13, 61.54, 57.27, 50.95, 40.08, 39.09, 30.96, 28.33, 26.44.

**2.5. Synthesis of chromophore BHG2 and BHG4**

Compound 4a or 4b (1.2 mmol) and CF_3_-TCF receptor (0.45 g, 1.4 mmol) were dissolved in 20 mL of absolute dry ethanol, refluxed at 80 °C for 0.5 h, and the solvent was removed by rotary evaporation. The crude product was purified by silica gel column chromatography and eluted with ethyl acetate/n-hexane (1:10 to 1:5) to obtain chromophore BHG2 and BHG4 with a yield of 38.2% and 48.2%, respectively. HRMS (ESI) (M+H)^+^ for BHG2: calcd for (C_101_H_97_F_3_N_4_O_6_Si_2_+H)^+^: 1575.6972; found: 1575.6934. HRMS (ESI) (M+H)^+^ for BHG4: calcd for (C_103_H_71_F_13_N_4_O_6_+H)^+^: 1707.5239; found: 1707.5278.

^1^H NMR for BHG2 (400 MHz, CDCl_3_) δ 7.59 (d, *J* = 7.5 Hz, 4H), 7.39 – 7.36 (m, 5H), 7.25 – 7.11 (m, 10H), 7.10 – 7.06 (dd, *J* = 13.3, 5.7 Hz, 6H), 6.86 (dd, *J* = 18.4, 8.5 Hz, 8H), 6.68 – 6.50 (m, 12H), 6.52 (d, *J* = 14.7 Hz, 1H), 3.94 (t, *J* = 5.4 Hz, 4H), 3.68 (d, *J* = 5.1 Hz, 4H), 3.51 (s, 3H), 2.16 (s, 2H), 2.02 (s, 2H), 0.87 (s, 3H), 0.80 (s, 21H), 0.14 (s, 12H). ^13^C NMR for BHG2 (101 MHz, CDCl_3_) δ 175.76, 162.54, 157.31, 154.45, 152.09, 151.88, 151.28, 148.52, 146.75, 140.05, 138.92, 138.47, 137.99, 135.72, 131.42, 130.18, 129.75, 129.55, 129.34, 129.23, 127.74, 127.43, 126.86, 126.14, 125.69, 123.75, 120.81, 120.22, 120.09, 119.57, 116.96, 114.13, 112.24, 111.72, 111.40, 110.90, 65.29, 64.32, 61.77, 57.94, 53.55, 51.09, 40.15, 39.33, 31.19, 31.03, 28.60, 28.06, 25.77, 18.14, -4.38.

^1^H NMR for BHG4 (400 MHz, CDCl_3_) δ7.74 (d, *J* = 7.5 Hz, 4H), 7.56 – 7.48 (m, 5H), 7.41 – 7.22 (m, 17H), 7.10 (dd, *J* = 16.1, 8.3 Hz, 8H), 6.83 – 6.66 (m, 12H), 5.03 (s, 4H), 4.11 (s, 4H), 3.85 (s, 4H), 3.65 (s, 3H), 2.43 – 2.22 (m, 4H), 0.98 (d, *J* = 23.9 Hz, 6H). ^13^C NMR for BHG4 (101 MHz, CDCl_3_) δ 175.60, 162.49, 157.23, 156.71, 152.05, 151.52, 151.01, 147.88, 146.99, 146.59, 144.50, 139.94, 139.32, 138.50, 137.50, 135.24, 131.32, 130.01, 129.65, 129.31, 129.23, 127.73, 127.45, 126.75, 125.92, 123.68, 120.20, 116.96, 114.33, 114.09, 112.61, 111.54, 111.23, 110.73, 110.36, 110.19, 110.02, 96.11, 95.80, 65.05, 64.14, 61.67, 57.98, 57.29, 51.29, 40.02, 39.18, 31.05, 28.47, 27.93.

**2.6. The film preparation process**

The chromophore BHG1-BHG4 were doped into the PMMA with the chromophore content of 25 wt% and were dissolved in 1,1,2-trichloroethane, respectively. Chromophores BHG2 and BHG4 were dissolved also in the same solvent. After ultrasonic dissolution of four solutions, filter them through 0.22 µm Teflon needle filter and spin-coated on an indium oxide (ITO) glass or silicon waveguide chip. The films were dried at 50 ℃ in the vacuum overnight to remove the residual solvents.

**2.7. The poling process of the high-speed EO modulator**

The pre-poling voltage is set to 20 V until the temperature reaches 138 ℃ (the poling temperature, T_p_). After the temperature reaches T_p_, the poling voltage gradually increases to 60 V, the chip rapidly drops to room temperature. The entire poling process lasted about 15 min.

**2.8. Characterization of the high-speed EO modulator**

For the V_π_ of the EO modulator, an arbitrary waveform generator is used to provide a triangular wave electrical signal loaded onto the modulator chip using a low frequency probe, and the output optical signal is converted into an electrical signal by photodetector, and the input and output signal are compared in real time by an oscilloscope to obtain the half-wave voltage Vπ of modulator chip.

For the 3 dB bandwidth of the EO modulator, the RF high frequency from VNA port 1 is loaded into the modulator chip by the RF probe, and the optical signal output from the modulator and enter the broadband photodetector, and the electrical signal output by the PD go back to VNA port 2 to obtain the S_21_ curve of the chip.

**3. ^1^H NMR and ^13^C NMR pictures of four chromophores BHG1-BHG4**


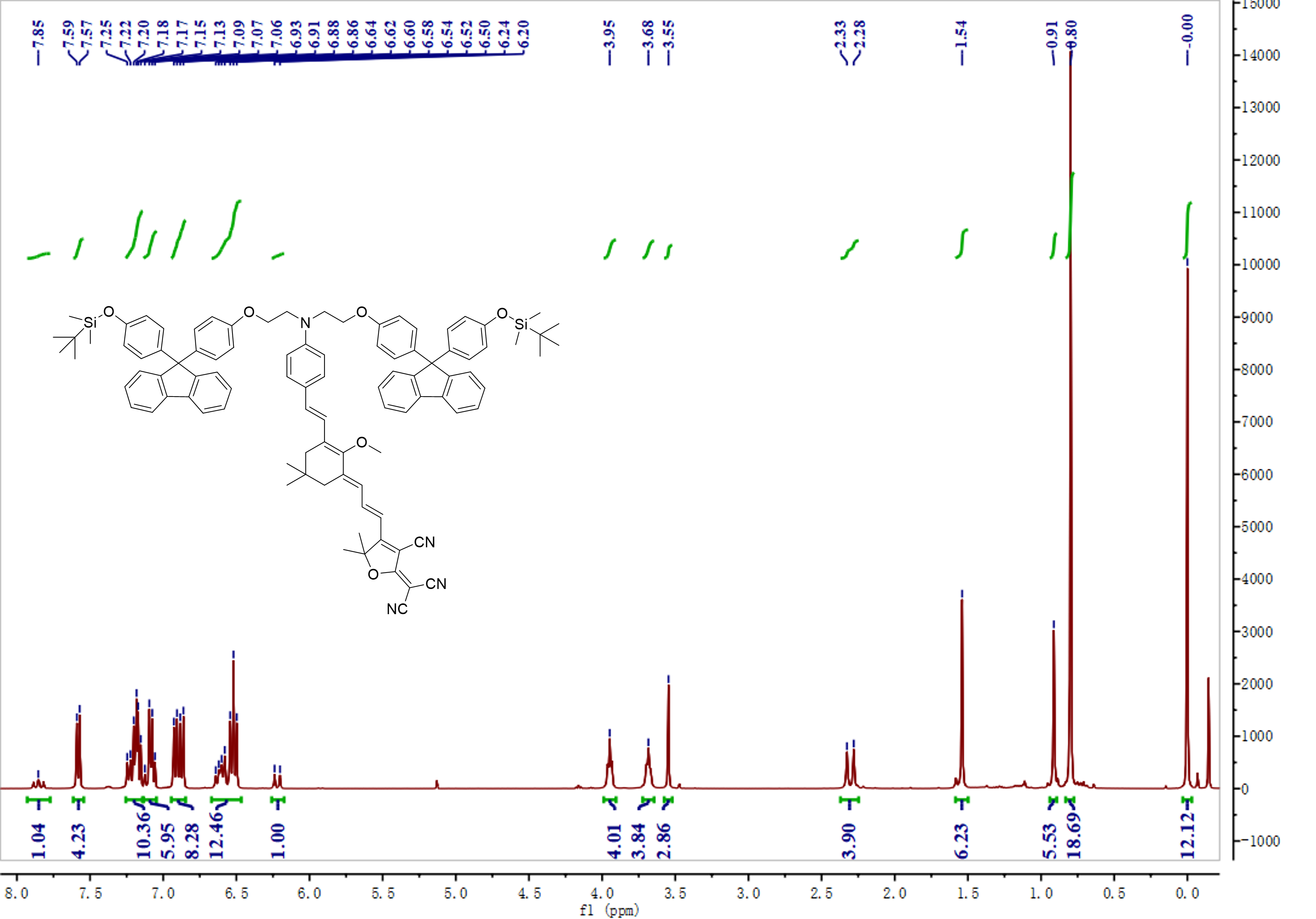


**Figure S1.** ^1^H-NMR spectrum of chromophore BHG1


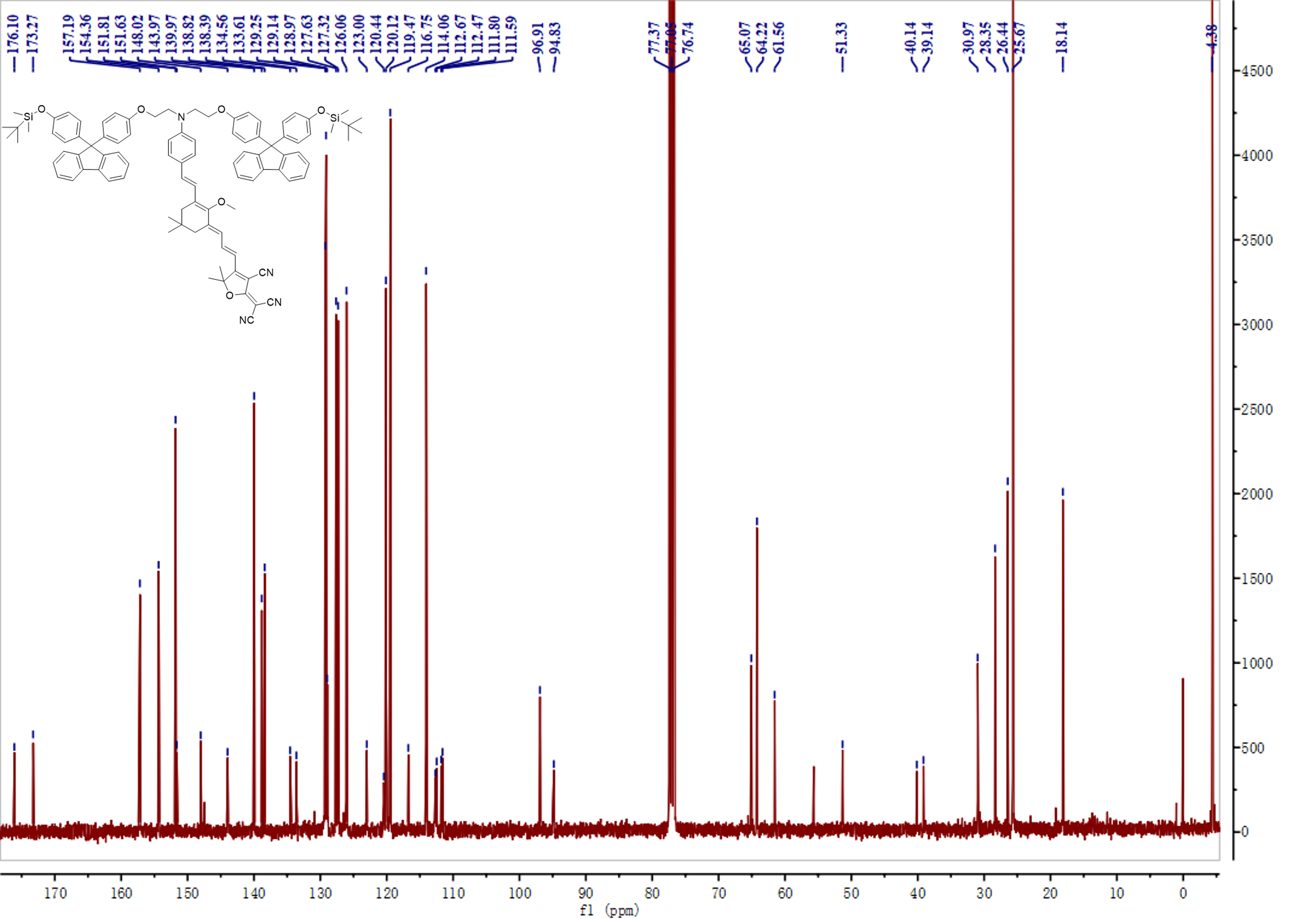


**Figure S2.** ^13^C-NMR spectrum of chromophore BHG1


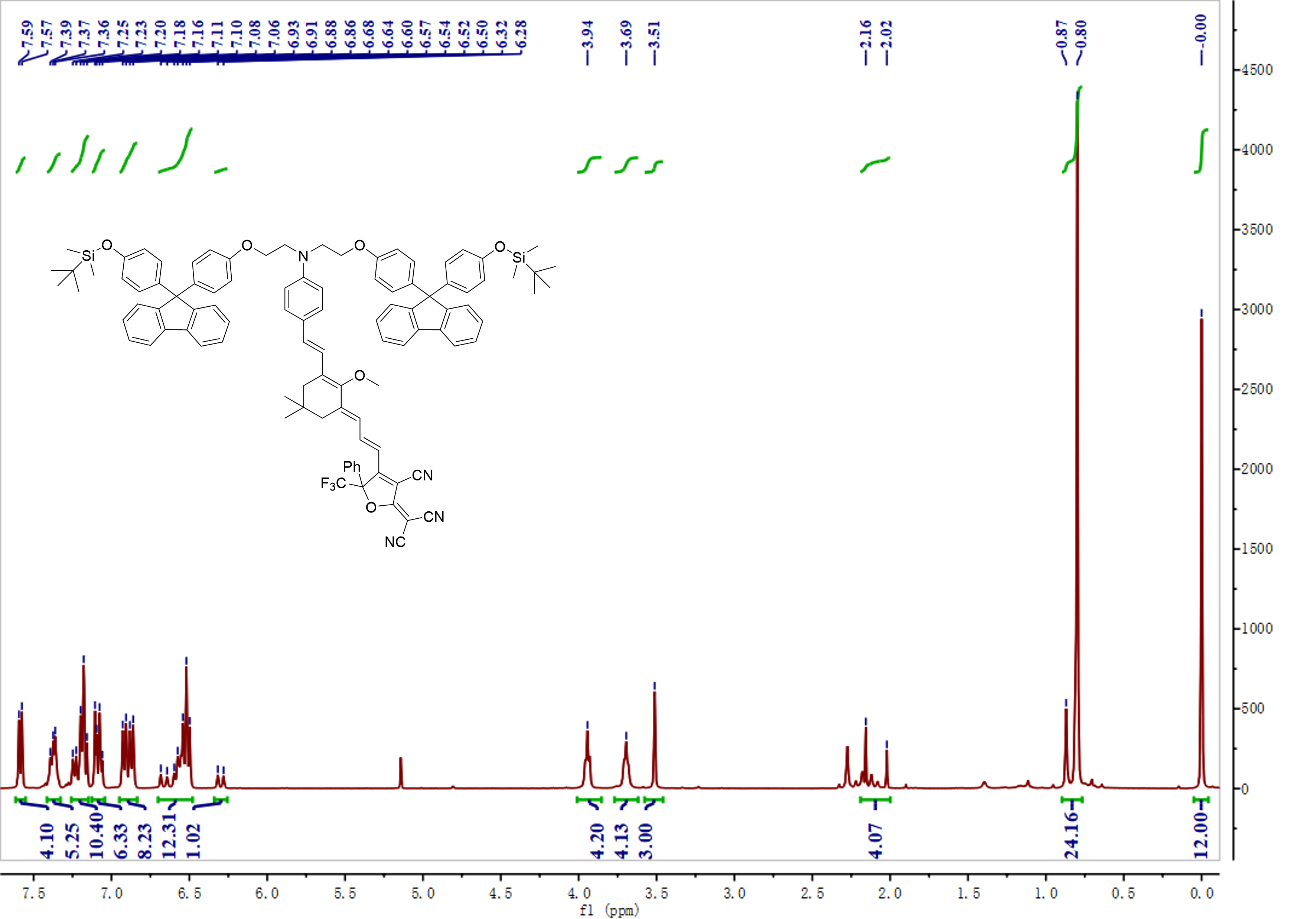


**Figure S3.** ^1^H-NMR spectrum of chromophore BHG2


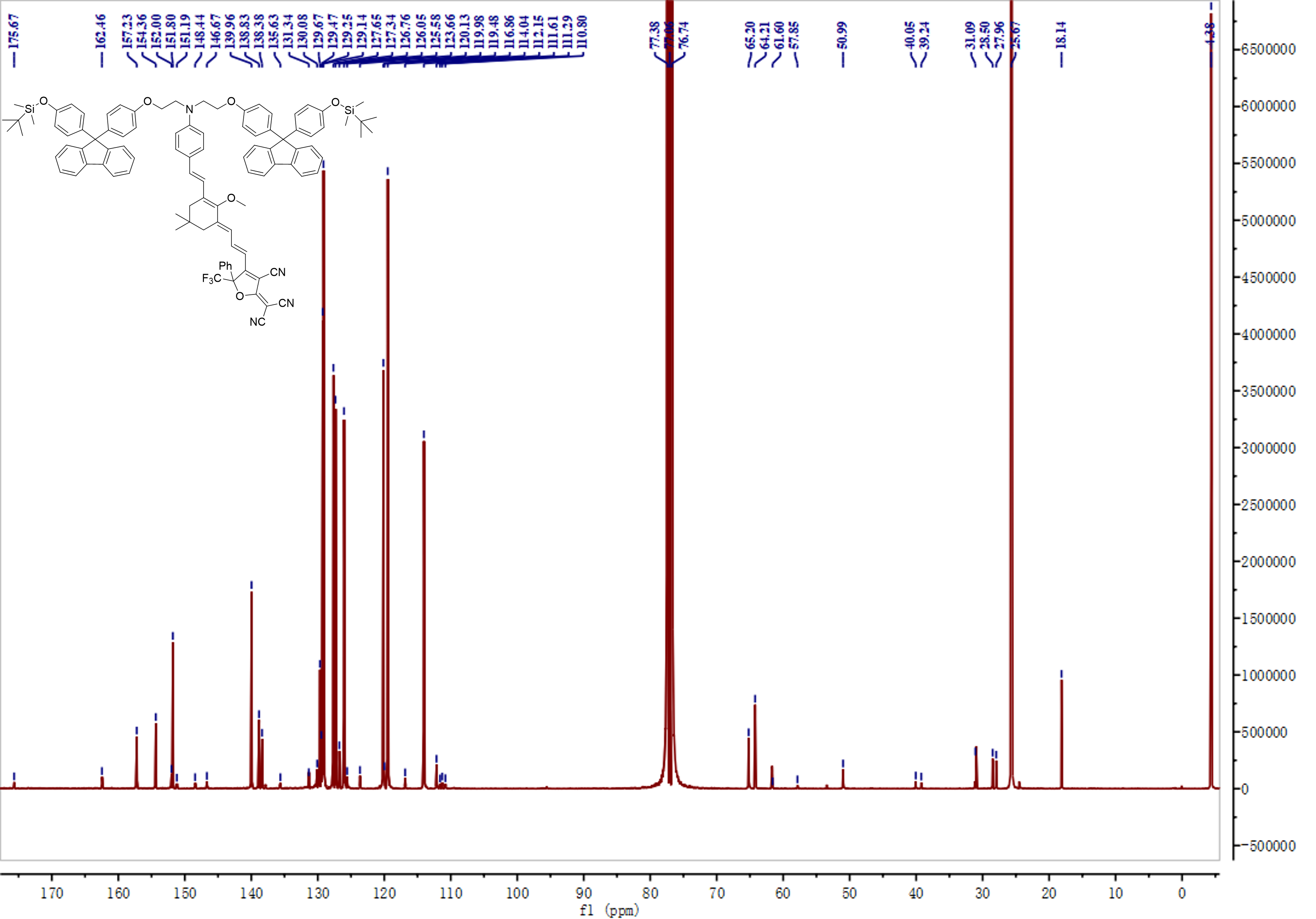


**Figure S4.** ^13^C-NMR spectrum of chromophore BHG2


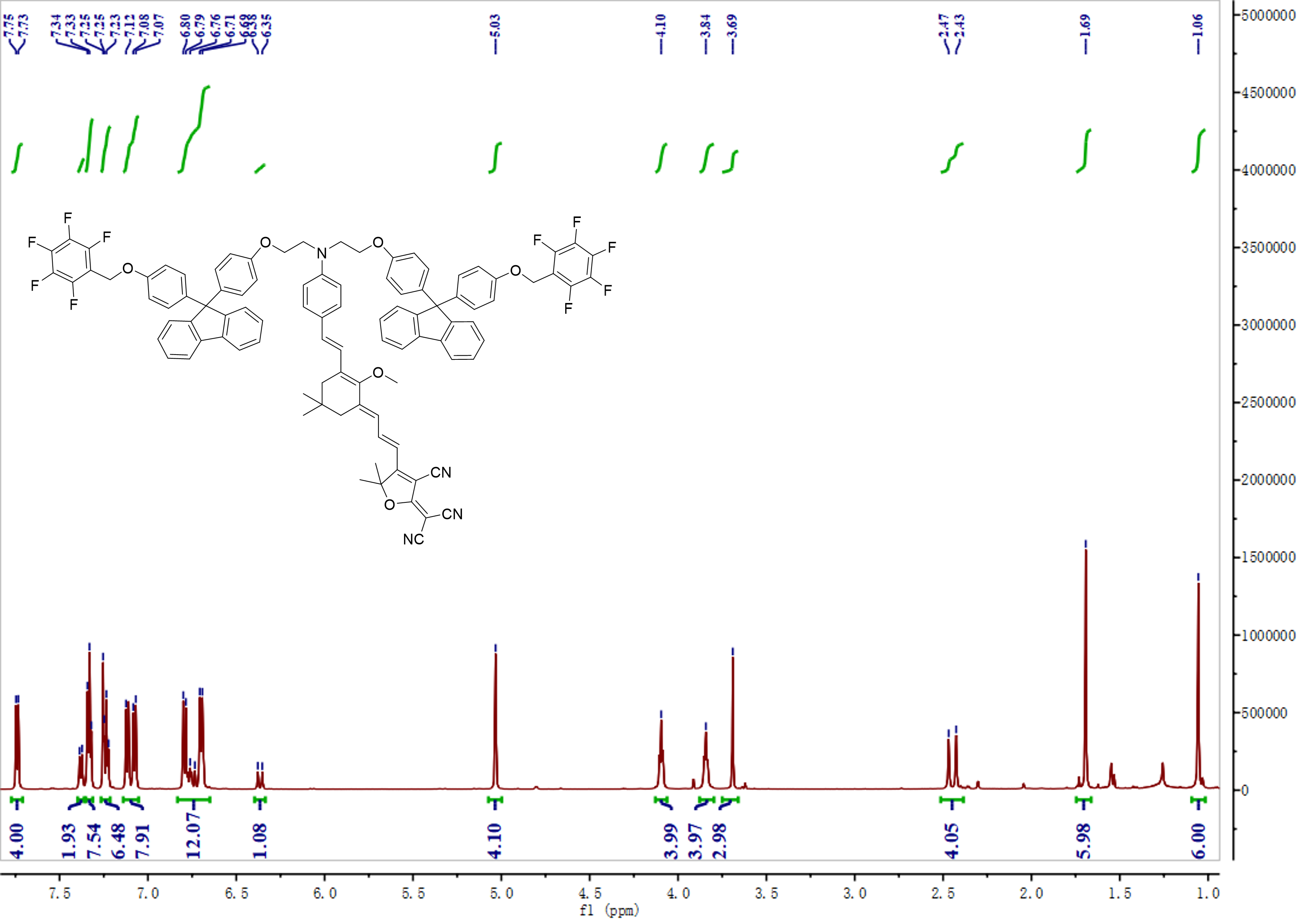


**Figure S5.** ^1^H-NMR spectrum of chromophore BHG3


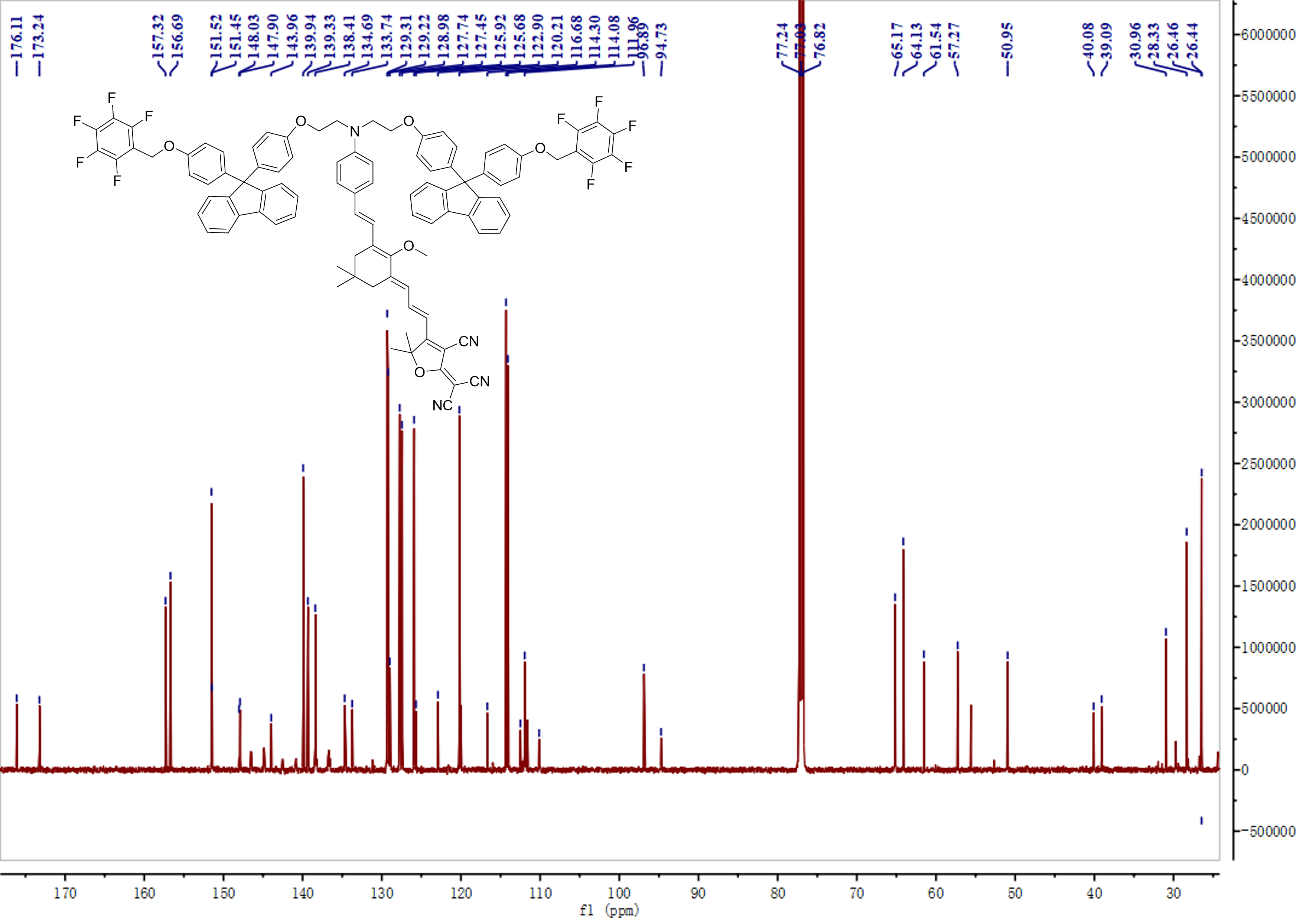


**Figure S6.** ^13^C-NMR spectrum of chromophore BHG3


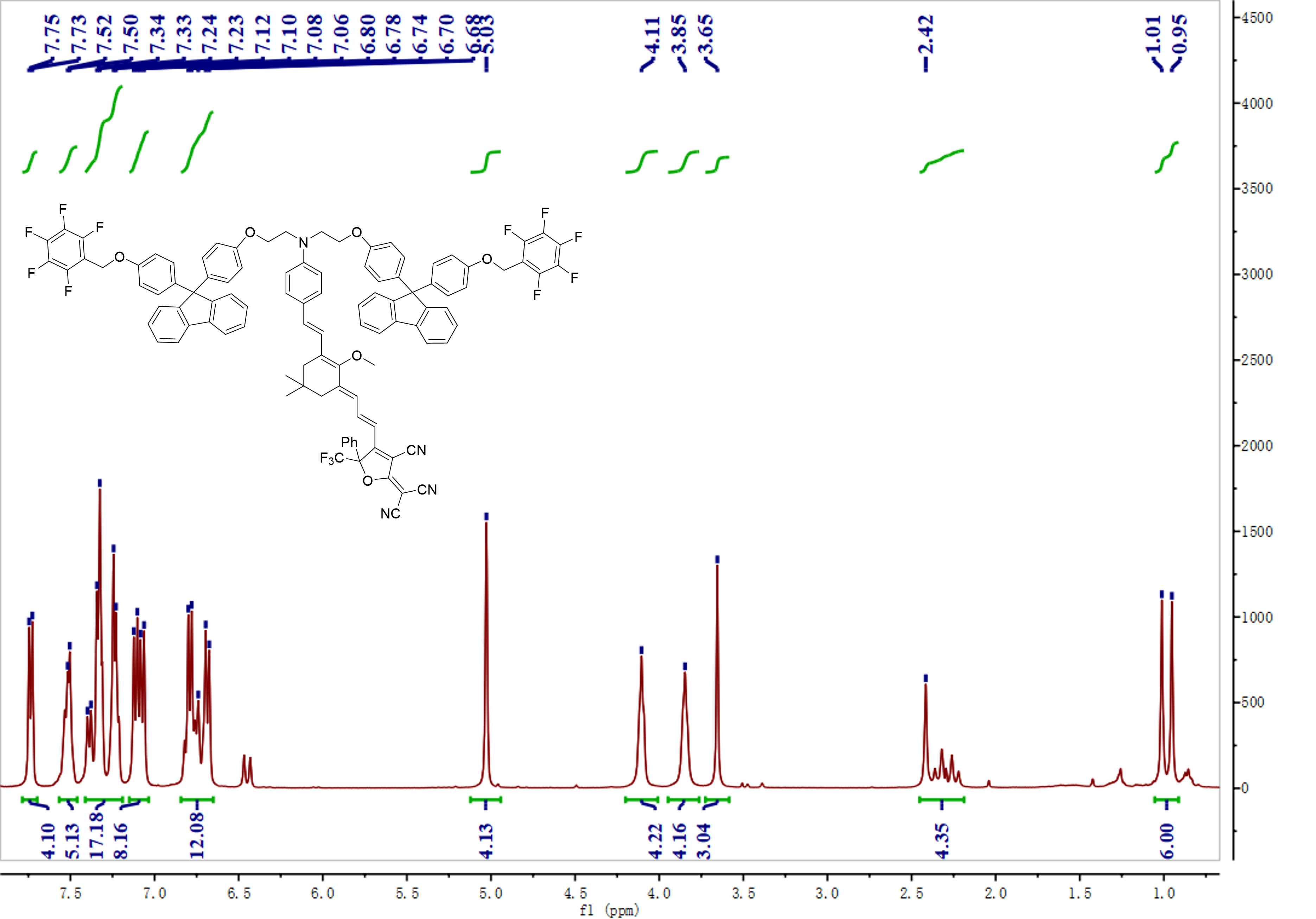


**Figure S7.** ^1^H-NMR spectrum of chromophore BHG4


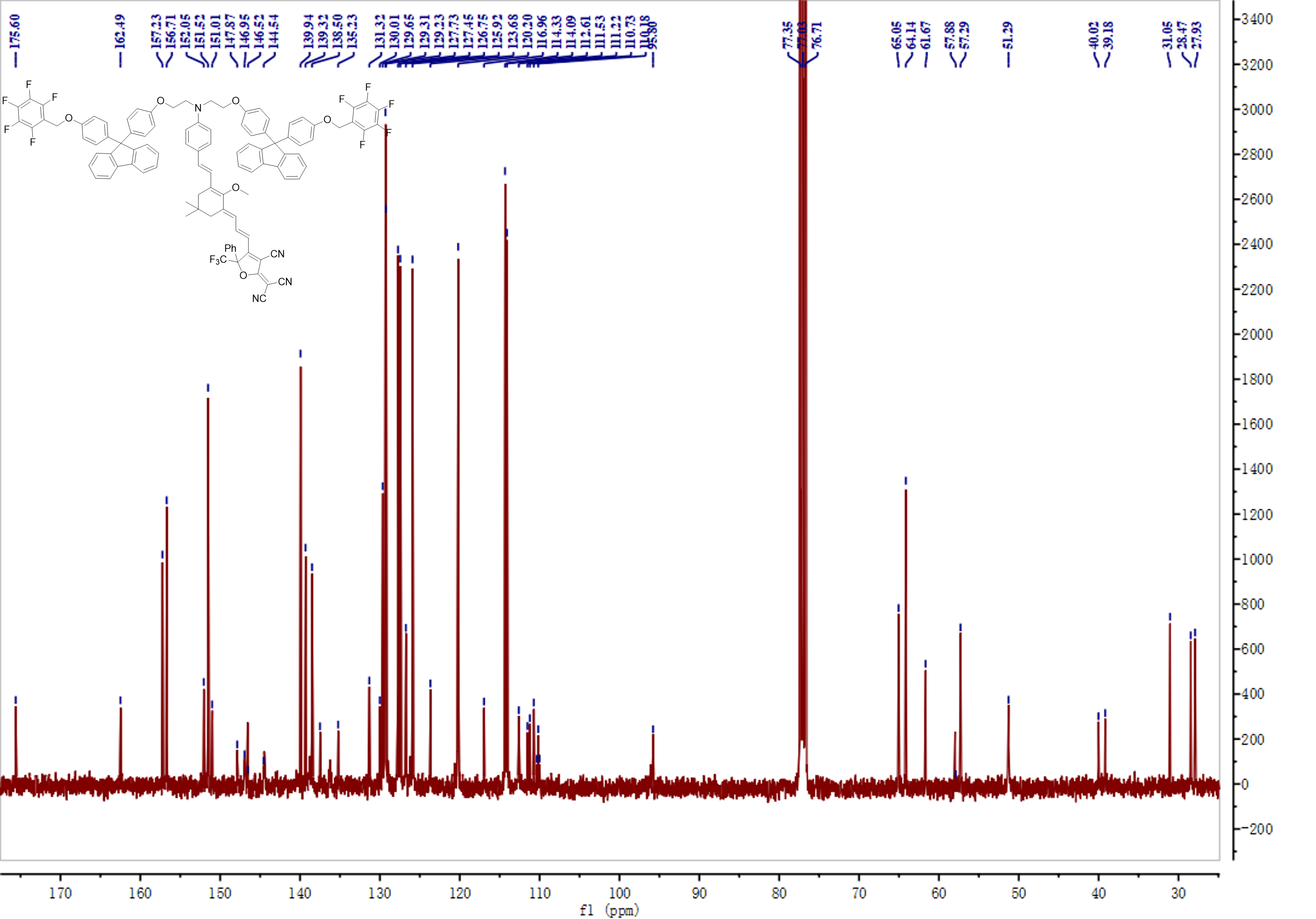


**Figure S8.** ^13^C-NMR spectrum of chromophore BHG4

1. **The absorption coefficients of BHG1-BHG4 in UV-Vis spectra.**

**Table S1.** The values of the absorption coefficients of four chromophores.

| Cmpd | Properties | acetone | CH_3_CN | CHCl_3_ | 1,4-dioxane | THF | toluene |
| --- | --- | --- | --- | --- | --- | --- | --- |
| BHG1 | ε/10^4^M^-1^cm^-1^ | 5.46 | 5.97 | 5.87 | 6.01 | 5.82 | 4.80 |
| BHG2 | ε/10^4^M^-1^cm^-1^ | 5.63 | 5.71 | 6.78 | 6.18 | 6.02 | 6.28 |
| BHG3 | ε/10^4^M^-1^cm^-1^ | 5.44 | 5.56 | 6.12 | 5.92 | 6.14 | 5.00 |
| BHG4 | ε/10^4^M^-1^cm^-1^ | 5.54 | 5.69 | 6.37 | 5.91 | 5.37 | 4.75 |

**5. The HOMO-LUMO orbital diagram of chromophores BHG1-BHG4**

**
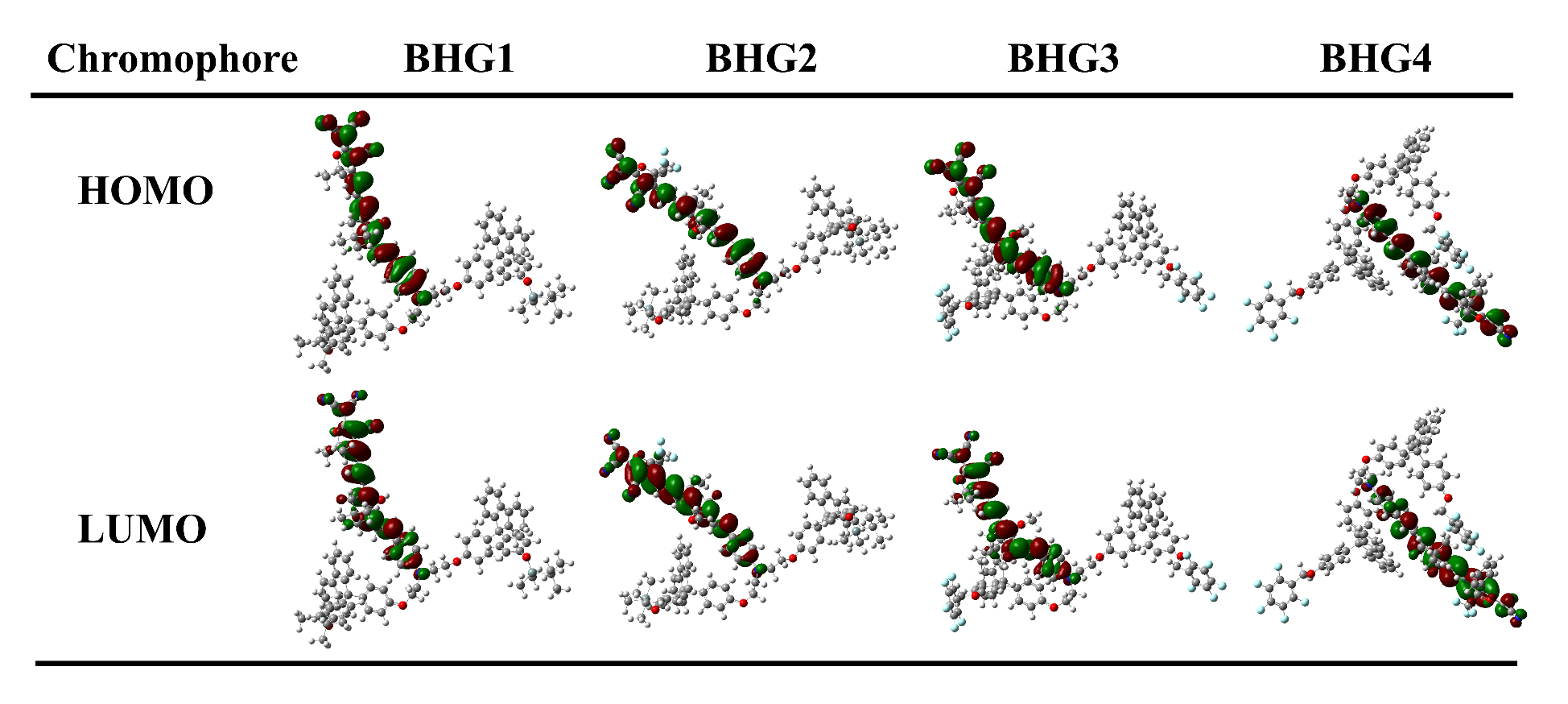
**

**Figure S9.** The HOMO-LUMO orbital diagram of chromophores BHG1-BHG4

**6. The self-forming film properties of the chromophores BHG1-BHG4**


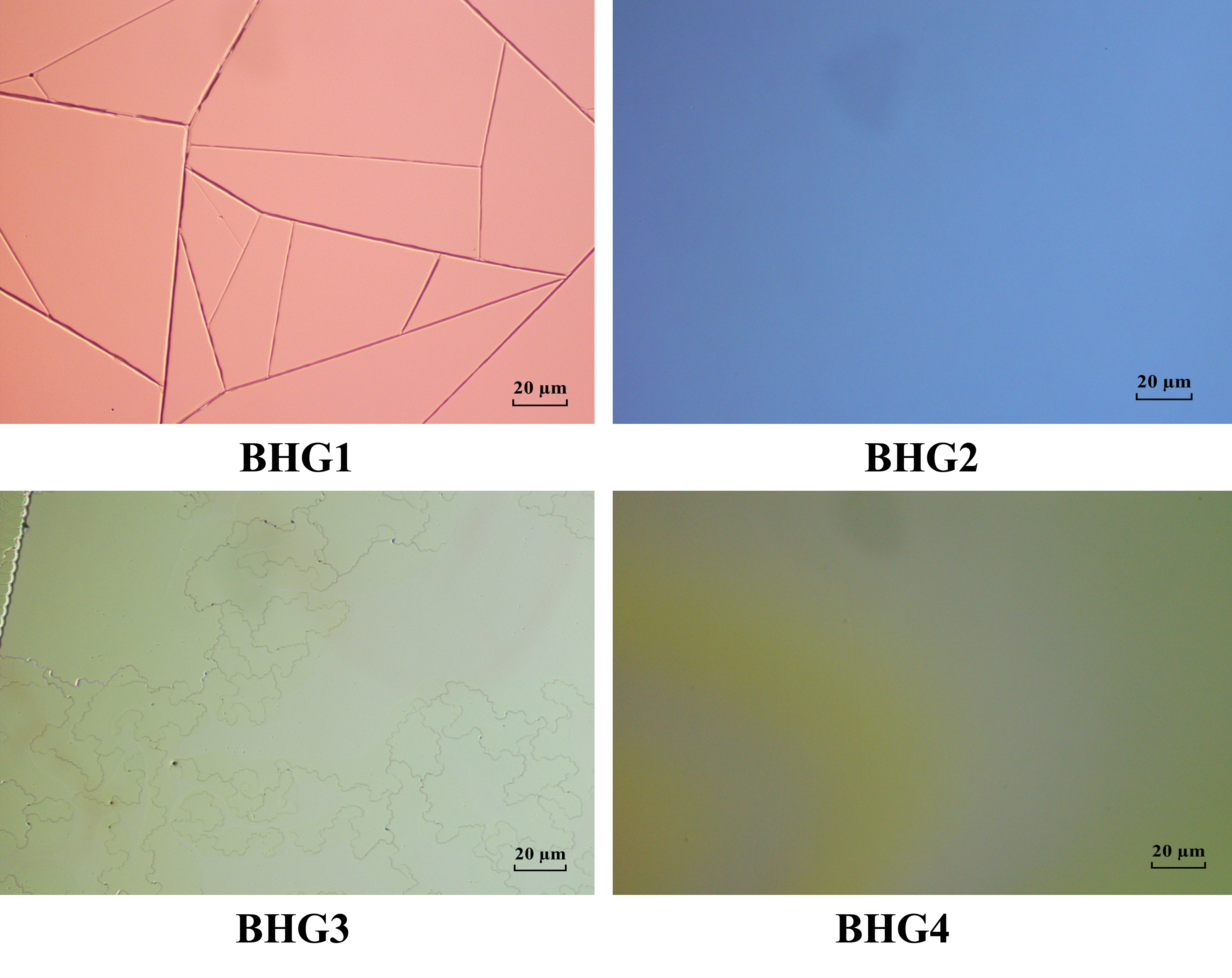


**Figure S10.** Photographs of the neat BHG1-BHG4 film under the optical microscope

**
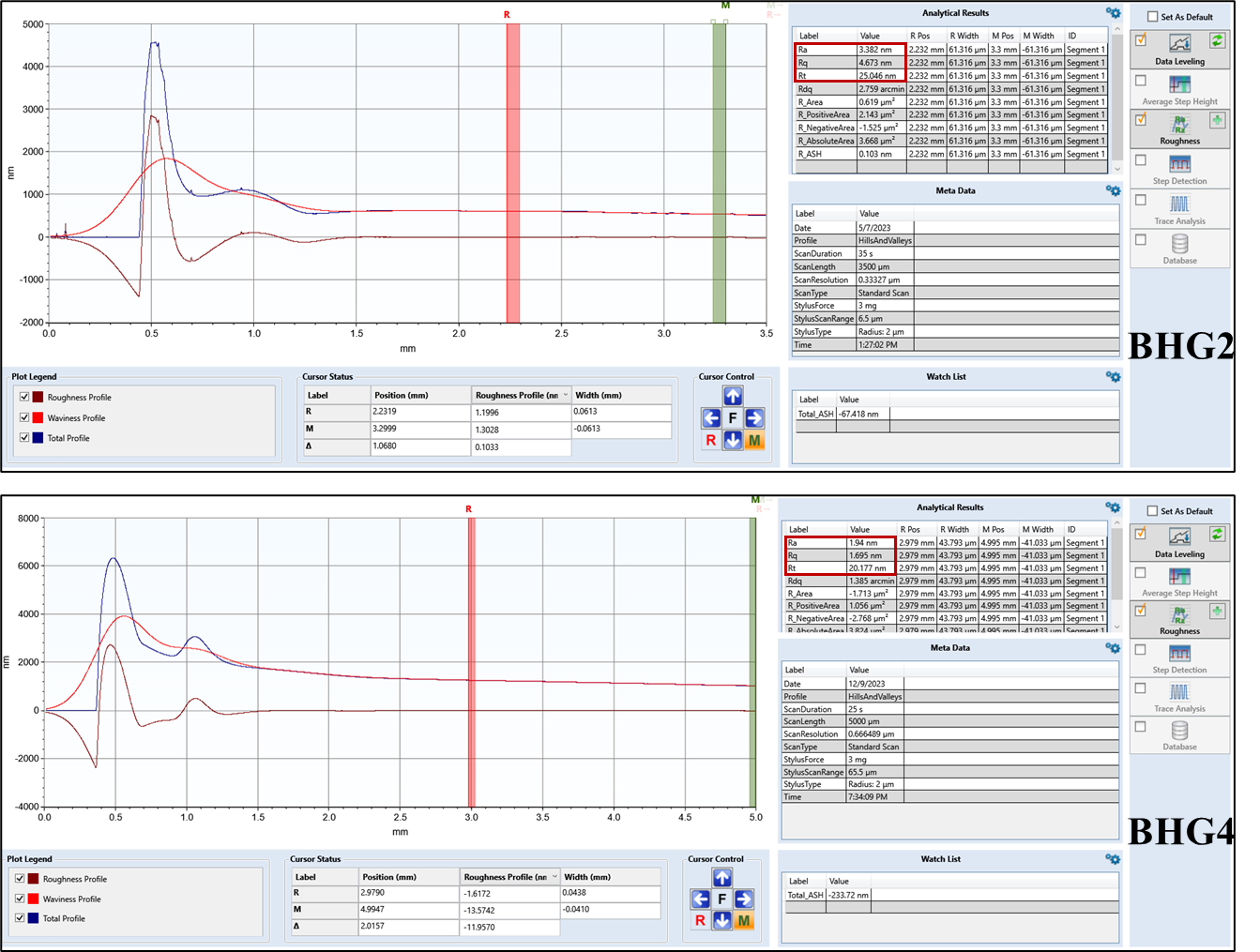
**

**Figure S11.** The roughness test results of the neat BHG2 and BHG4 films

**Table S2.** The roughness data of the neat BHG2 and BHG4 films

| Chromophore | Ra（nm） | Rq（nm） | Rt（nm） |
| --- | --- | --- | --- |
| BHG2 | 3.38 | 4.67 | 25.05 |
| BHG4 | 1.94 | 1.70 | 20.18 |

**7. Poling curve plots of r_33_ versus poling field**


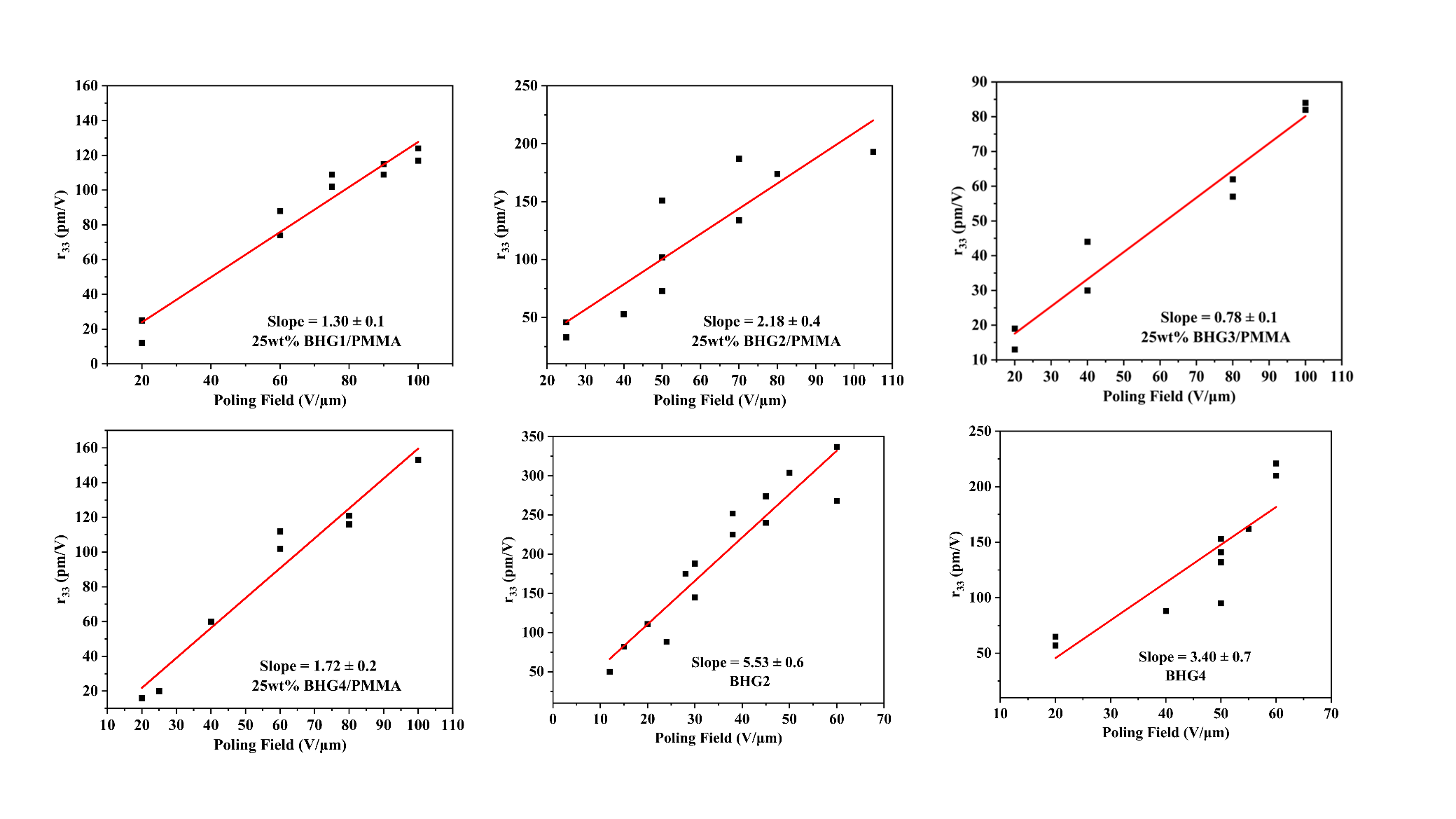


**Figure S12.** Poling curve plots of r_33_ versus poling field.

**8.** **Long-term thermal stability of the neat BHG2 and JRD1 film**

**
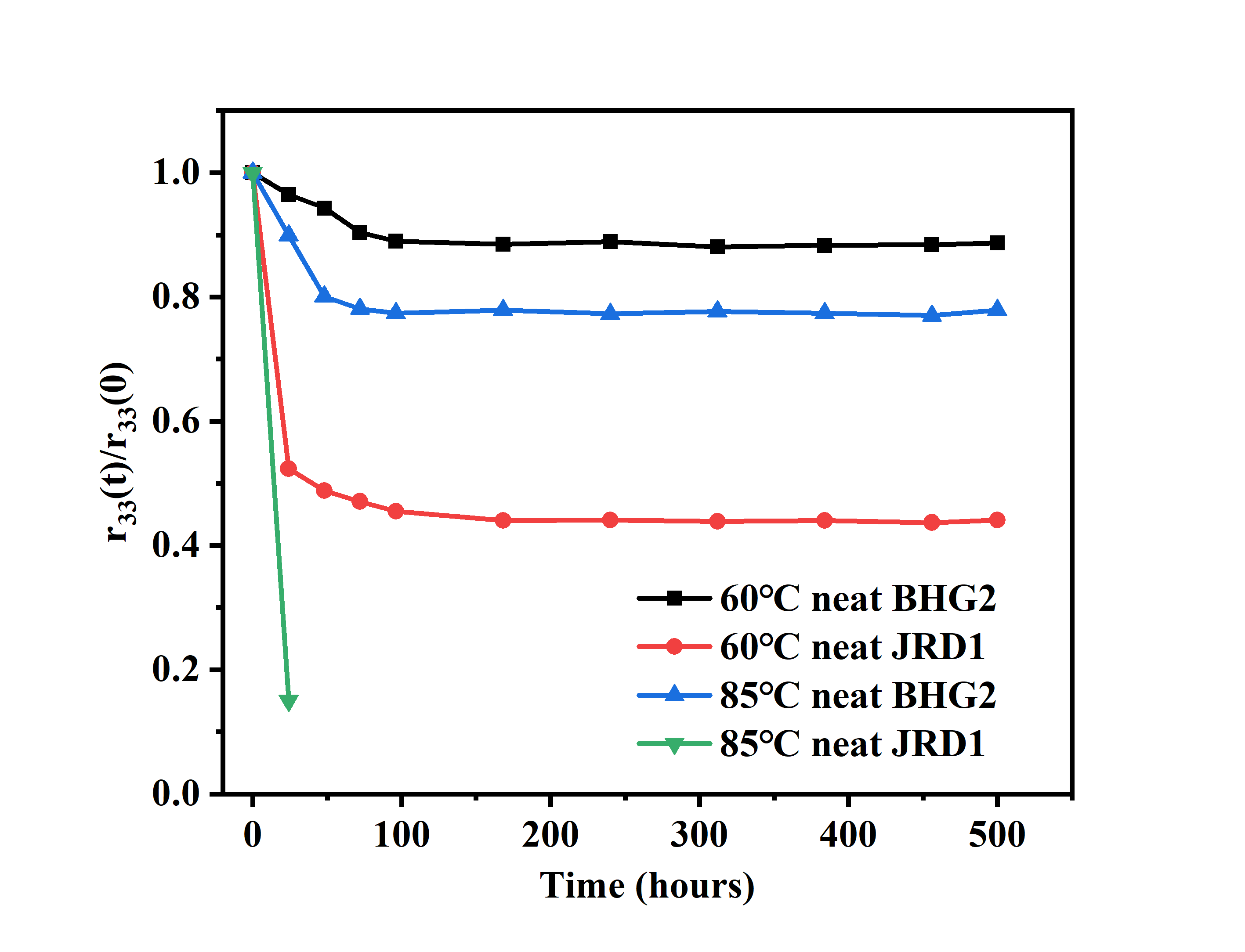
**

**Figure S13.** Long-term thermal stability of the neat BHG2 and JRD1 film at the 60 ℃ and

85 ℃.

**9. The refractive index n and extinction coefficient k of the neat BHG2 film**

Organic electro-optic material is the key material of electro-optic modulator, and the refractive index of the film is an important parameter for the EO materials. The refractive index n and extinction coefficient k of the neat BHG2 film before and after poling are tested by ellipsometer in the wavelength range of 400 ~1700 nm and shown in Figure S14 and Table S3, which provides the necessary parameters for the design of optical waveguide devices.

The neat BHG2 film shows a large k value in the visible light region, indicating that the material has a large absorption loss in this region. Among them, for the application of electro-optic modulators, the optical communication band C-band (1530 ~1565 nm) and O-band (1260 ~1310 nm) are very important. The refractive index n of the neat BHG2 film before and after poling at 1550 nm is 1.747 and 1.725, and n value after poling has decreased.^2^ The same situation will also occur at 1310 nm. The extinction coefficient k of the neat BHG2 film before and after poling at 1550 nm is 2.69×10^-4^ and 5.64×10^-4^, which illustrates there is a slight increase in loss after poling.


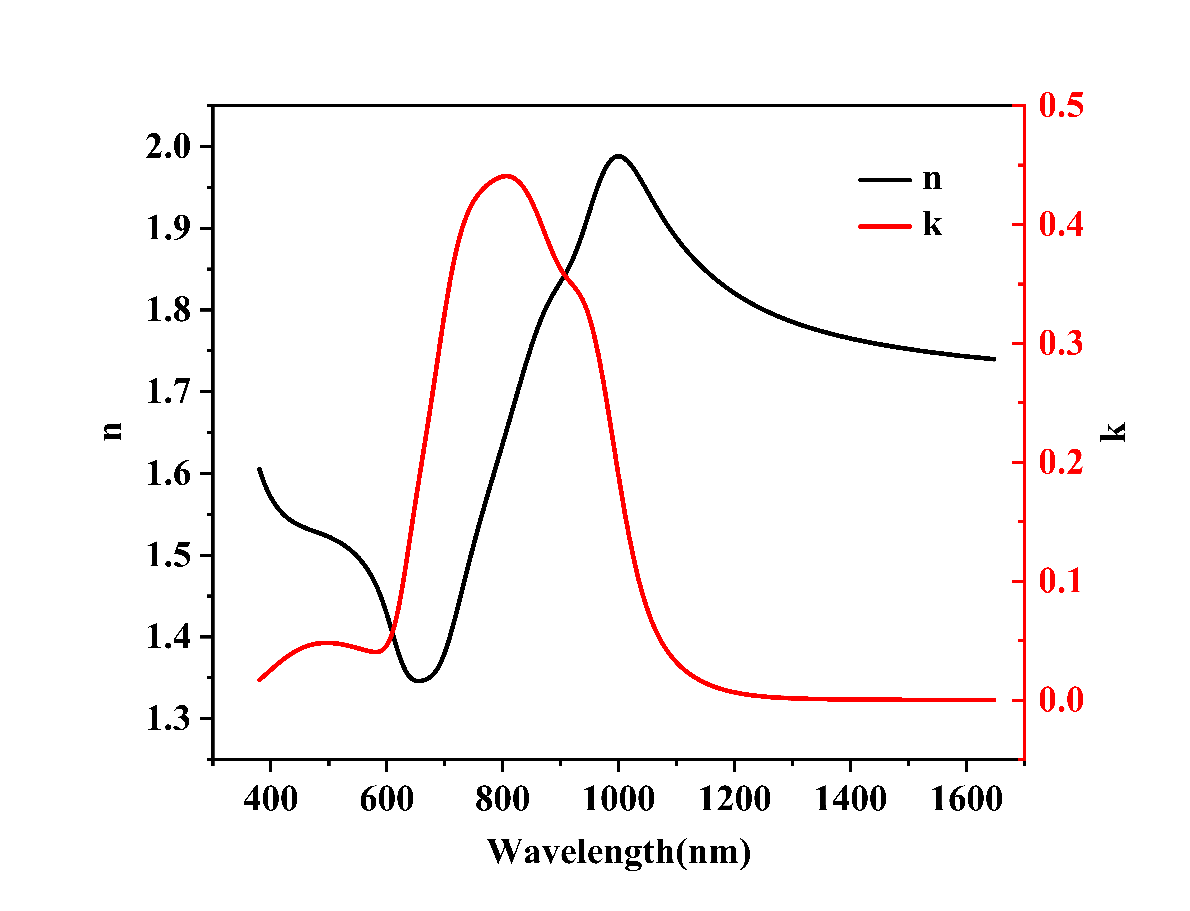


**Figure S14.** The refractive index n and extinction coefficient k of the neat BHG2 film before poling.

**Table S3.** The refractive index n of extinction coefficient k of BHG2 film before and after poling at 1550 nm and 1310 nm

| BHG2 | n  before poling | k  before poling | n  after poling | k  after poling |
| --- | --- | --- | --- | --- |
| 1310 nm | 1.783 | 1.40×10^-3^ | 1.761 | 6.47×10^-3^ |
| 1550 nm | 1.747 | 2.69×10^-4^ | 1.725 | 5.64×10^-4^ |

**10. Testing setup for the EO frequency response of the modulator**

The EO S_21_ curve was tested using the follow setup in Figure S15. Firstly, utilize a laser to couple the input coupler of the modulator with a single-mode optical fiber, and the output optical fiber is then linked to a high-frequency detector. Subsequently, a high-frequency probe is placed in contact with the GSG electrode, while another high-frequency electrode is connected to a 50-ohm load impedance. After establishing these connections, link the high-frequency probe to a vector network analyzer and connect the detector to the same vector network analyzer.


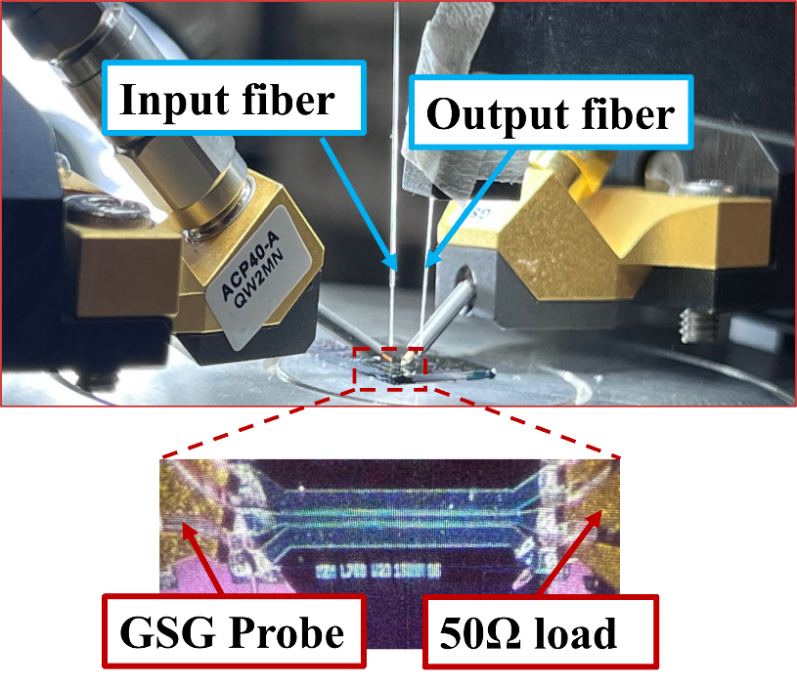


**Figure S15.** Testing setup for the EO frequency response of the modulator including electrical connections (probe) and optical paths using single mode fibers.

**References**

1. M. He, T. M. Leslie and J. A. Sinicropi, α-Hydroxy ketone precursors leading to a novel class of electro-optic acceptors. *Chem. Mater*. **2002**, *14*, 2393-2400.
